# Recommender System for the Efficient Treatment of COVID-19 Using a Convolutional Neural Network Model and Image Similarity

**DOI:** 10.3390/diagnostics12112700

**Published:** 2022-11-05

**Authors:** Madhusree Kuanr, Puspanjali Mohapatra, Sanchi Mittal, Mahesh Maindarkar, Mostafa M. Fouda, Luca Saba, Sanjay Saxena, Jasjit S. Suri

**Affiliations:** 1 Department of Computer Science and Engineering, IIIT, Bhubaneswar 751003, India; 2 Stroke Monitoring and Diagnostic Division, AtheroPointTM, Roseville, CA 95661, USA; 3 Department of Electrical and Computer Engineering, Idaho State University, Pocatello, ID 83209, USA; 4 Department of Radiology, University of Cagliari, 09123 Cagliari, Italy; 5 Knowledge Engineering Center, Global Biomedical Technologies, Inc., Roseville, CA 95661, USA

**Keywords:** recommender system, COVID-19, CNN model, feature extraction, ResNet-50, Maxwell–Boltzmann similarity

## Abstract

***Background***: Hospitals face a significant problem meeting patients’ medical needs during epidemics, especially when the number of patients increases rapidly, as seen during the recent COVID-19 pandemic. This study designs a treatment recommender system (RS) for the efficient management of human capital and resources such as doctors, medicines, and resources in hospitals. We hypothesize that a deep learning framework, when combined with search paradigms in an image framework, can make the RS very efficient. ***Methodology***: This study uses a Convolutional neural network (CNN) model for the feature extraction of the images and discovers the most similar patients. The input queries patients from the hospital database with similar chest X-ray images. It uses a similarity metric for the similarity computation of the images. ***Results***: This methodology recommends the doctors, medicines, and resources associated with similar patients to a COVID-19 patients being admitted to the hospital. The performance of the proposed RS is verified with five different feature extraction CNN models and four similarity measures. The proposed RS with a ResNet-50 CNN feature extraction model and Maxwell–Boltzmann similarity is found to be a proper framework for treatment recommendation with a mean average precision of more than 0.90 for threshold similarities in the range of 0.7 to 0.9 and an average highest cosine similarity of more than 0.95. ***Conclusions***: Overall, an RS with a CNN model and image similarity is proven as an efficient tool for the proper management of resources during the peak period of pandemics and can be adopted in clinical settings.

## 1. Introduction

SARS-CoV-2 coronavirus was first discovered and reported in Wuhan, China, in 2019 and has spread globally, causing a health hazard [1,2,3]. On 30 January 2020, the World Health Organization labelled the outbreak a Public Health Emergency of International Concern, and on 11 March 2020, it was declared a pandemic. COVID-19 has varied effects on different people. The majority of infected patients experience mild to moderate symptoms and do not require hospitalization. Fever, exhaustion, cough, and a loss of taste or smell are all common COVID-19 symptoms [4]. Loss of smell, confusion, trouble breathing or shortness of breath, and chest discomfort are some of the major symptoms that lead to serious pneumonia in both lungs [1,4,5,6]. COVID-19 pneumonia is a serious infection with a high mortality rate. The signs of a COVID-19 infection progressing into dangerous pneumonia include a fast pulse, dyspnea, confusion, rapid breathing, heavy sweating, and pulmonary embolism [7,8]. It induces serious lung inflammation, as seen in lung microscopy [9]. It puts strain on the cells and tissue that cover the lungs’ air sacs. The oxygen for breathing is collected and supplied to the bloodstream through these sacs. Due to injury, tissue breaks off and blocks the lungs [10]. The sacs’ walls might thicken, making breathing extremely difficult.

**Figure 1:**
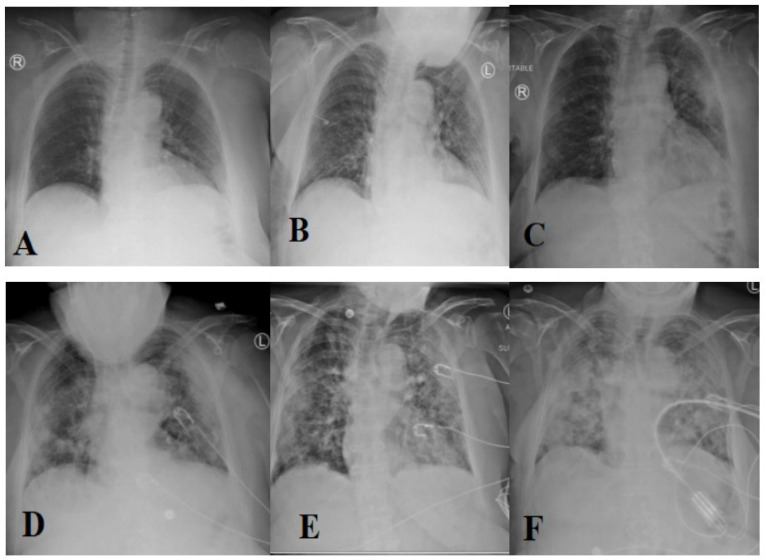
Evolution of an X-ray image for a COVID-19 patient over days (1 (**A**), 3 (**B**), 6 (**C**), 7 (**D**), 8 (**E**), and 10 (**F**), respectively) [11] (reproduced with permission).

The most prevalent method of diagnosing individuals with respiratory disorders is chest radiography imaging [11,12,13]. At the beginning of COVID-19, a chest radiography image appeared normal, but it gradually altered in a fashion that may be associated with pneumonia or acute respiratory distress syndrome (ARDS) [11]. Figure 1 depicts the progression of chest X-ray images for a 45-year-old person infected with COVID-19. Roughly 15% of COVID-19 patients require hospitalization and oxygen therapy. Approximately 5% of people develop serious infections and require a ventilator.

During the peak period of infection transmission, having enough oxygen and a ventilator is also a major challenge for hospitals [14,15]. As a result, hospitals and medical practitioners are under a lot of stress trying to deal with critical patients who have been admitted to hospitals [16]. They concentrate on providing good care to individuals who are hospitalized so that the mortality rate can be lowered, and the patients can recover quickly. However, hospitals’ capability to provide adequate treatments to hospitalized patients is sometimes limited by the availability of doctors and resources. In this scenario, a recommender system (RS) using machine learning (ML) approaches might be used to administer the best treatment while working with limited resources [17,18]. As the mortality rate and recovery rate of seriously hospitalized COVID-19 patients generally depend upon the amount of infection in the lungs [19,20,21], the radiographic lung images of those patients can be used to recommend proper treatment in terms of a doctor, medicine, and other related resources.

From the perspective of the RS’s implementation, a new patient’s chest X-ray image is sent to the proposed system, and doctors, medicines, and resources are recommended for that patient. The proposed system assumes that the database consists of lung images and other information such as the name of the doctor assigned, medicines, and resources provided, such as intensive care unit (ICU), oxygen therapy, and ventilators. The COVID-19 patients who were admitted to the hospital in the past successfully recovered from the hospital. It uses a collaborative filtering method to find similar COVID-19 patients to new COVID-19 patients using image similarity. The proposed approach uses convolutional neural networks (CNN) for feature extraction [22,23,24] from images and utilizes those feature vectors for similarity computation.

**Figure 2:**
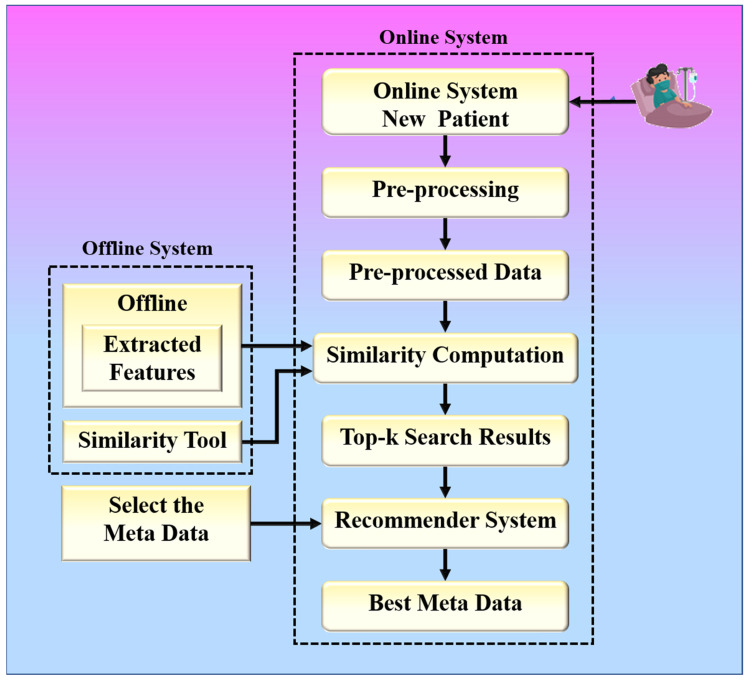
The global treatment recommender system for COVID-19.

The proposed collaborative RS uses image similarity to produce recommendations, as image similarity is a popular and efficient technique in image-based RS [25,26,27]. Traditional image-based RS recommends images for a given input query image. The novelty of the proposed RS is that it recommends some metadata information such as doctors, medicines, and resources for a given input query such as a chest X-ray image. The proposed system is built around two hypotheses. The first hypothesis states that the proposed system’s performance is dependent on the feature extraction technique used by CNN models. The second hypothesis states that the proposed system’s performance is also affected by the similarity measure used for similarity computation. Higher similarity assures a more accurate recommendation. The chest X-ray images are compared based on their feature vectors. The CNN model is used to effectively extract feature vectors from chest X-ray images. The combination of a robust search strategy and the best feature selection approach may make the RS more powerful for efficient and accurate recommendations. Figure 2 represents the global system representation of the proposed approach. The proposed system can be analyzed as a combination of an online and an offline system. The offline system is responsible for the feature extraction process from the images, and the online system handles the recommendation process. The web-based system is equipped with a performance module that calculates accuracy based on the known reference values in the test dataset. 

In particular, the objectives of this article include: (i) to propose an efficient RS system for COVID-19 based on chest X-ray images to address the impact of an RS on the efficient handling of situations in hospitals during the peak period of a pandemic with limited resources; (ii) to use multiple CNN models to construct an RS using COVID-19 chest X-ray images; (iii) to propose a unique design by embedding four kinds of search paradigm in the CNN-based framework; (iv) comparative data analysis of different similarity measure in the RS framework, providing metadata which includes doctors, medicines, and resources; (v) finally, to mitigate the impact of an RS in the healthcare domain through improved services and efficient resource management.

The remainder of this study is as follows: Section 2 includes the discussion of RS, CNN, the feature extraction process, measures for similarity computation, and studies related to the proposed work. The proposed model is explained in Section 3, and the experimental evaluation is discussed in Section 4. Section 5 focuses on future scope, and the article is concluded in Section 4.

## 2. Background Literature

Many researchers have presented various models employing traditional machine learning approaches in the past for the identification of COVID-19 using radiography images [2,28]. Zimmerman et al. [29] reviewed many cardiovascular uses of machine learning algorithms, as well as their applications to COVID-19 diagnosis and therapy. The authors in refs. [30,31] proposed image analysis tools to classify lung infection in COVID-19 based on chest X-ray images and claimed that artificial intelligence (AI) methods have the potential to improve diagnostic efficiency and accuracy when reading portable chest X-rays. In ref. [19], the authors established an ensemble framework of five classifiers such as K-nearest neighbors (KNN), naive Bayes, decision tree, support vector machines (SVM), and artificial neural network for the detection of COVID-19 using chest X-ray images. Ref. [32] describes a method for detecting SARS-CoV-2 precursor-miRNAs (pre-miRNAs) that aids in the identification of specific ribonucleic acids (RNAs). The method employs an artificial neural network and proposes a model with an estimated accuracy of 98.24%. The proposed method would be useful in identifying RNA target regions and improving recognition of the SARS-CoV-2 genome sequence in order to design oligonucleotide-based drugs against the virus’s genetic structure.

Due to the unprecedented benefits of a deep CNN in image processing, it has been successfully utilized by various researchers for the identification and accurate diagnosis of COVID-19. In ref. [20], the authors proposed a deep learning (DL) model for the detection of COVID-19 by annotating computed tomography (CT) and X-ray chest images of patients. In ref. [33], various DL models such as ResNet-152, VGG-16, ResNet-50, and DenseNet-121 were applied to radiographic medical images for the identification of COVID-19 and were compared and analyzed. To overcome the lack of information and enhance the training time, the authors also applied transfer learning (TL) techniques to the proposed system. A voting-based approach using DL for the identification of COVID-19 was proposed in ref. [34]. The proposed method makes use of CT scan chest images of patients and utilizes a voting mechanism to classify a CT scan image of a new patient. Various DL algorithms for identifying COVID-19 infections from lung ultrasound imaging were reviewed and compared by the authors in ref. [35]. The proposed method adopts four pre-trained models of DL such as InceptionV3, VGG-19, Xception, and ResNet50, for the classification of a lung ultrasound image. In ref. [36], the authors compared the results of using CNNs pre-trained with ML-based classification algorithms. The major purpose of this research was to see how CNN-extracted features affect the construction of COVID-19 and non-COVID-19 classifiers. The usefulness of DL learning algorithms for the detection of COVID-19 using chest X-ray images is demonstrated in ref. [37]. The proposed approach was implemented using 15 different pre-trained CNN models, and VGG-19 showed a maximum classification accuracy of 89.3%. In ref. [38], an object detection approach using DL for the identification of COVID-19 in chest X-ray images was presented. The suggested method claims a sensitivity of 94.92% and a specificity of 92%.

Many kinds of research have also been conducted in the past using image segmentation, image regrouping, and other hybrid techniques for accurate diagnosis of COVID-19 [39]. In ref. [40], the authors proposed an innovative model using multiple segmentation methods on CT scan chest images to determine the area of pulmonary parenchyma by identifying pulmonary infiltrates (PIs) and ground-glass opacity (GGO). In ref. [41], the authors proposed a hybrid model for the detection of COVID-19 using feature extraction and image segmentation techniques to improve the classification accuracy in the detection of COVID-19. In ref. [42], a hybrid approach of feature extraction and CNN on chest X-ray images for the detection of COVID-19 using a histogram-oriented gradient (HOG) algorithm and watershed segmentation methodology was proposed. This proposed hybrid technique showed satisfactory results in the detection of COVID-19 with an accuracy of 99.49%, sensitivity of 93.65%, and specificity of 95.7%. In ref. [43], the authors came up with a new way to determine COVID-19 in images of chest X-rays using image segmentation and image regrouping. The proposed approach was found to outperform the existing models for the identification of COVID-19 in terms of classification accuracy with a lower amount of training data. In ref. [44], the transfer learning technique was used in conjunction with image augmentation to train and validate several pretrained deep Convolutional Neural Networks (CNNs). The networks were trained to classify two different schemes: (i) normal and COVID-19 pneumonia and (ii) normal, viral, and COVID-19 pneumonia with and without image augmentation. The classification accuracy, precision, sensitivity, and specificity for both schemes were 99.7%, 99.7%, 99.7%, and 99.55% and 97.9%, 97.95%, 97.9%, and 98.8%, respectively. The high accuracy of this computer-aided diagnostic tool can significantly improve the speed and accuracy of COVID-19 diagnosis. A systematic and unified approach for lung segmentation and COVID-19 localization with infection quantification from CXR images was proposed in ref. [45] for accurate COVID-19 diagnosis. The proposed method demonstrated exceptional COVID-19 detection performance, with sensitivity and specificity values exceeding 99%.

RS has also been useful in combating the COVID-19 pandemic by making recommendations such as medical therapies for self-care [46], wearable gadgets to prevent the COVID-19 outbreak [47], and unreported people to reduce infection rates by contact tracing [48], among others. An RS based on image content was proposed in ref. [25] that employed a random forest classifier to determine the product’s class or category in the first phase and employed the JPEG coefficients measure to extract the feature vectors of the photos in the second phase to generate recommendations using feature vector similarity. A neural network-based framework for product selection based on a specific input query image was provided by ref. [26]. The suggested system employed a neural network to classify the supplied input query image, followed by another neural network that used the Jaccard similarity measure to find the most comparable product image to that input image. In ref. [27], the authors developed a two-stage DL framework using a neural network classifier and a ranking algorithm for recommending fashion images based on similar input images. Traditional RS frequently faces a significant challenge in learning relevant features of both users and images in big social networks with sparse relationships between users and images, as well as the widely different visual contents of images. Refs. [49,50,51] presented a strategy for solving this data sparsity problem in content-based and collaborative filtering RS by importing additional latent information to identify users’ probable preferences.

The majority of previous research in RS based on computer vision was conducted for the e-commerce domain, with only a few works carried out for the healthcare domain, according to the literature. It was also revealed from the literature that image similarity is one of the successful techniques used for designing RS in computer vision. Furthermore, the efficacy of computer vision in RS in providing solutions for combating the COVID-19 pandemic has yet to be investigated. In this context, we suggest a health recommender system (HRS) that uses image similarity and collaborative filtering to provide treatment suggestions for COVID-19.

## 3. Methodology

### 3.1. Recommender System

**Figure 3:**
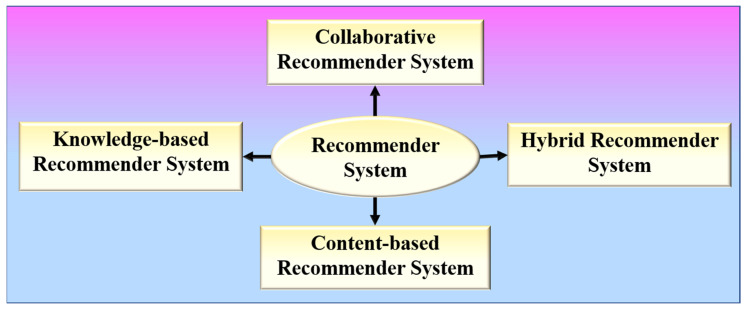
Various types of recommender systems.

RS is a software program that aids in the personalization of users and is based on the principle of information filtering [52]. RS has the following formal definition: Let P represent the set of all users, and Q represent the set of all items that can be recommended. Let t be a utility function that measures the usefulness of item q to user p, i.e., t: p × q → S, where S is an ordered set. The items q′ ∈ Q that maximize the user’s utility will then be recommended for each user p ∈ P. As a result, ∀p ∈ P, q_p_′ = arg max_q∈Q_ t (p, q) may be stated more formally. RS can be broadly divided into four types, as shown in Figure 3.

Content-based RS or cognitive RS provides recommendations based on a comparison of the items’ content with a user profile [53,54]. Collaborative RS collects preferences or taste information from the collaborated users to produce automatic predictions regarding the user’s interests [55,56]. Memory-based and model-based are the two different categories of a collaborative RS. A memory-based collaborative RS makes use of all the data to provide recommendations based on user or item similarity, whereas model-based collaborative filtering RS entails creating a model based on all of the data in order to detect similarities between users or items for recommendation purposes. Hybrid RS combines two or more recommendation algorithms in different ways to take advantage of their different strengths [57,58]. A knowledge-based RS intelligently filters a group of targets to fulfil the user’s preferences. It assists in overcoming the difficulties of both collaborative and content-based RSs [59,60].

An RS used in health applications to analyze patients’ digital data and filter out the best information according to their profile is known as a health recommender system (HRS) [61,62]. HRS can be thought of as a decision-making system that plays a big role in society by advising patients on suitable disease treatments and doctors on good disease diagnoses.

### 3.2. Convolutional Neural Network

**Figure 4:**
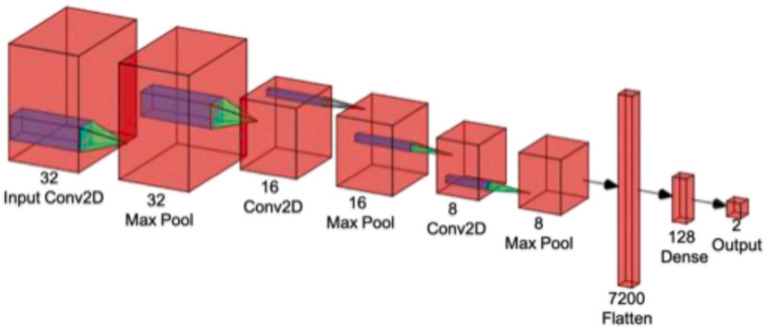
The basic architecture of convolutional neural network [22] (reproduced with permission).

A CNN is a powerful DL tool for image processing and recognition [63]. The fundamental architecture of a CNN contains three distinct types of layers: convolutional, pooling, and fully connected, as shown in Figure 4.

#### 3.2.1. Convolutional Layer

The basic layer of CNN is the convolutional layer, which has the responsibility of extracting features and recognizing patterns in input images. The CNN extracts low-level and high-level features by passing images from the training dataset through a filter comprised of feature maps and kernels [23]. The convolutional layer’s output can be expressed in the following way:

(1) $$q_{s}^{r}\left(m,n\right)=\sum_{c}^{\;}\sum_{x,y}^{\;}p_{c}\left(x,y\right)g_{r}^{s}\left(u,v\right),\;$$

where *q_s_^r^* (*m*, *n*) is the convolution layer, and *p_c_* (*x*, *y*) is an element of the input image tensor *p_c_*, multiplied by the $g_{r}^{s}\left(u,v\right)$ index of the *s*th convolutional kernel *s_r_* of the *r*th layer element-wise.

#### 3.2.2. Pooling Layer

The pooling layer, or the down-sampling layer, gathers comparable data in the vicinity of the feature layer and generates the dominating response inside this layer. The pooling process aids in the extraction of a group of features that are invariant to *x_i_*, and it can be defined as Equation (2).

*Y_r_^S^* = *t_j_* (*Q^S^_r_*), (2)

where for the *s*th input feature-map *Q^s^_r_*, *Y_r_^S^*conveys the pooled feature-map of the *r*th layer.

#### 3.2.3. Fully Connected Layer

The fully connected layer is utilized for classification at the end of the CNN network. This layer takes the features that have been collected at different stages of the network as the input and then analyses and compares those features to the results from all the other layers.

#### 3.2.4. Activation Function

The activation function modifies the weighted sum input of one node for a given layer and uses it to activate that node for a certain input. The activation function assists in the learning of feature patterns by acting as a decision function. *ReLU* is one of the most widely used activation functions due to its ability to handle the gradient problem in CNN models. Mathematically, the *ReLU* activation function can be defined as follows:

(3) $$ReLU\left(x\right)=\left\{\begin{array}{l} \;0,\;\;x<0\\ x,\;\;x\geq \;0 \end{array}\right.$$

#### 3.2.5. Batch Normalization

Batch normalization is used for the normalization of the output of the preceding layers, which can assist with issues such as internal covariance shifts in feature maps. The equation for the batch normalization of transformed feature map *Q_r_^s^* can be defined as shown in Equation (4).

(4) $$B_{r}{}^{s}=\frac{Q_{r}{}^{s}-{µ}_{b}}{\sqrt{\sigma_{b}{}^{2}+\in }}$$

where $B_{r}{}^{s}$ denotes the normalized feature map, and $Q_{r}{}^{s}$ represents the input feature map. The mean and the variance of the feature map are represented by *µ_b_* and *σ_b_^2^*, respectively. *ϵ* is used to deal with the numerical instability caused by division by zero.

#### 3.2.6. Dropout and Flatten Layer

Dropout is a technique for adding regularization to a CNN network, which establishes generalization by omitting some connections at random. After removing some random connections, the network design with the lowest weight value is chosen as a close approximation of all the suggested networks. The Flatten layer transforms the pooled feature map into a one-dimensional array that is passed as a single feature vector to the next layer.

### 3.3. Feature Extraction Methods

CNN is widely used in computer vision for feature extraction because it can discover relevant features from images without requiring human interaction and is computationally efficient. There are various models of CNN for the feature extraction process. In this study, we tested the performance of our proposed system with two specific CNN models, namely: the residual neural network (ResNet) and the visual geometry group (VGG). We used three different versions of ResNet, namely ResNet-50, ResNet-101, and ResNet-152, and two versions of VGG, namely VGG-16 and VGG-19. The detailed architectures of both ResNet and VGG are described in the following subsections.

#### 3.3.1. ResNet

**Figure 5:**
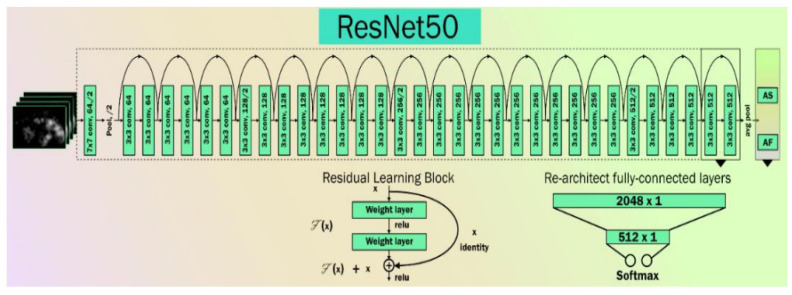
The basic architecture of ResNet.

ResNet is an artificial neural network that can solve the problem of training very deep networks using residual blocks [64]. Several COVID-19-related publications have been tried using ResNet or the Hybrid nature of ResNet [65,66,67]. The basic architecture of a ResNet network is shown in Figure 5.

**Figure 6:**
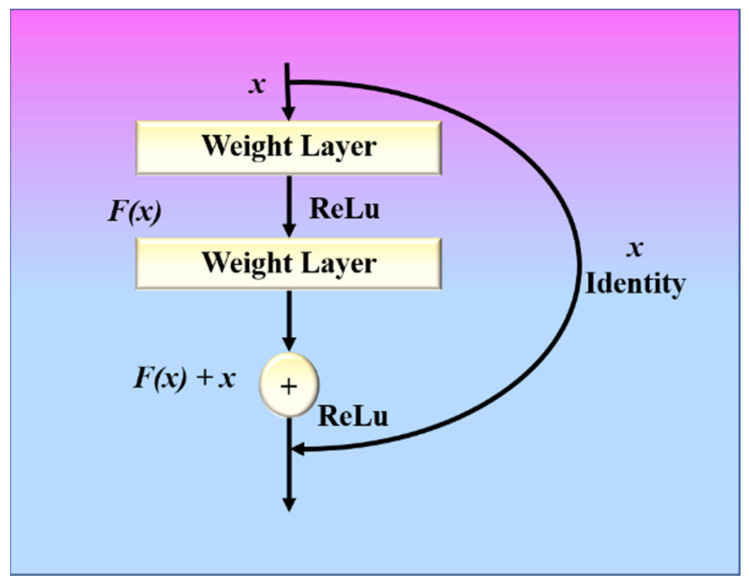
ResNet with residual block.

A ResNet model with these residual blocks is shown in Figure 6. A direct connection in the ResNet model can skip some layers and is known as a “skip connection”, which is the heart of the model. The model produces a different output due to this skipped connection. When the connection is not skipped, the input X is multiplied by the weights of the following layer, and a bias term is added to this. Therefore, Equation (5) can be used to describe the model’s output function.

(5) $$H\left(x\right)=\left\{\;\begin{array}{c} f\left(x+b\right),\;\;\;\;without\;skip\;connection\\ f\left(x\right),\;\;\;\;\;\;\;\;\;with\;skip\;connection \end{array}\right..$$

Double or triple-layer skips with nonlinearities (*ReLU*) and batch normalization are used in most ResNet models [64]. An additional weight matrix can be utilized, and such models are termed “Highway Nets.” We used three variations of ResNet, namely ResNet-50 [68], ResNet-101 [69], and ResNet-152 [70].

#### 3.3.2. VGG Net

**Figure 7:**
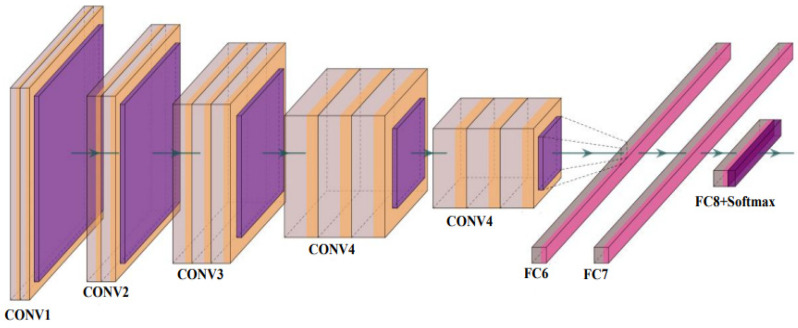
The basic architecture of VGG Net [73] (reproduced with permission); CONV: convolution layer and FC: fully connected network.

VGG Net is a traditional CNN model composed of blocks, each consisting of 2D convolution and max pooling layers. The basic architecture of a VGG Net is shown in Figure 7. It was created to improve the performance of a CNN model by increasing the depth of the model. The VGG16 and VGG-19 are the two versions available. VGG-16 and VGG-19 include 16 and 19 layers of convolution layers, respectively. The VGG Net architecture serves as the foundation for cutting-edge object recognition models. The VGG Net, which was created as a deep neural network (DNN), outperforms baselines on a variety of tasks and datasets. Small convolutional filters are used to build the VGG network. VGG-16 [71] and VGG-19 [72] are two different versions of VGG that we used to test our proposed system.

#### 3.3.3. Similarity Measures

The similarity measure is a means of determining how closely data samples are related. It plays an important role in computer vision by aiding in the comparison of two images by determining their feature vector similarity [64,74]. The proposed model uses the cosine similarity measure to compute the similarity between two feature vectors to find the most similar images to the input image, which are further utilized for the recommendation process.

#### 3.3.4. Cosine Similarity Measure

The similarity between two vectors using cosine similarity can be calculated as follows:

(6) $$\mathrm{CosSim}\left(U,\;V\right)=\frac{U\cdot V}{\left|\left|U\right|\right|\left|\left|V\right|\right|}=\frac{\sum_{i=1}^{n}U_{i}V_{i}}{\sqrt{\sum_{i=1}^{n}U_{i}{}^{2\;\;}}\sqrt{\sum_{i=1}^{n}V_{i}{}^{2\;\;}}}$$

where *U* and *V* represent two vector components. The cosine similarity is measured on a scale of 0 to 1, with 0 representing no similarity and 1 representing 100% similarity. All the other values in the range [0, 1] show the equivalent percentage of similarity.

#### 3.3.5. Euclidean Distance Similarity Measure

The Euclidean similarity between image vectors *U* and *V* can be calculated as follows:

(7) $$EucSim\left(U,\;V\right)=\sqrt{\sum_{i=1}^{n}{\left(U_{i}-V_{i}\right)}^{2}}.$$

The Euclidean similarity is also measured on a scale of 0 to 1, with 0 representing no similarity and 1 representing 100% similarity. All other values in the [0, 1] range reflect the equivalent percentage of similarity.

#### 3.3.6. Jaccard Similarity Measure

Jaccard similarity is a popular proximity measurement that is used to determine the similarity between two objects. The Jaccard similarity is calculated by dividing the number of observations in both sets by the number of observations in either set. It is also graded on a scale of 0 to 1, with 0 indicating no similarity and 1 indicating complete similarity. All other values in the [0, 1] range correspond to the same percentage of similarity. The similarity between two vectors using Jaccard similarity can be calculated as:

(8) $$JacSim\left(U,\;V\right)=\frac{\left|U\cap V\right|}{\left|U\cup V\right|}.$$

#### 3.3.7. Maxwell–Boltzmann Similarity Measure

The Maxwell–Boltzmann similarity is a popular similarity measure for document classification and clustering [75]. It is calculated using the overall distribution of feature values and the total number of nonzero features found in the documents. The difference between the two documents is represented by the following:

(9) $$D\left(U,\;V\right)\approx log\frac{N_{D}}{G_{D}},$$

where,

(10) $$N_{D}=\left\{\begin{array}{c} 0.5*\;\sum {\left(U_{k}-V_{k}\right)}^{2}\;\;\;\;\;\;\;\;\;\;\;if\;U_{k}\;and\;V_{k}>0\\ \lambda_{k}{}^{q}*0.5*\;\sum {\left(U_{k}\right)}^{2}\;\;\;\;if\;U_{k}>0\;and\;V_{k}=0\\ \lambda_{k}{}^{q}*0.5*\;\sum {\left(V_{k}\right)}^{2}\;\;\;\;if\;V_{k}>0\;and\;U_{k}=0\\ 0\;\;\;\;\;\;\;\;\;\;\;\;\;\;\;\;\;\;Otherwise \end{array}\right.$$

and

(11) $$G_{D}=\left\{\begin{array}{c} tnz\;\;\;\;\;\;\;\;\;\;\;\;\;\;\;\;\;\;\;\;\;\;\;if\;U_{k}\;and\;V_{k}>0\;\\ tnzu\;\;\;\;\;\;\;\;\;\;\;\;if\;U_{k}>0\;and\;V_{k}=0\\ tnzv\;\;\;\;\;\;\;\;\;\;\;if\;V_{k}>0\;and\;U_{k}=0\\ 0\;\;\;\;\;\;\;\;\;\;\;\;\;\;\;\;\;\;Otherwise \end{array}\right.,$$

where,

*tnz* = the total number of nonzero attributes,
*tnzu* = the total number of features of *U* having nonzero items,
*tnzv* = the total number of features of *V* having nonzero items,
0 < *λ* < 1, *k* denotes features, and *q* denotes the number of present–absent pairs.

The Maxwell–Boltzmann similarity is calculated from the value of *D* as follows:

(12) $$MaxwellBoltzSim\left(U,\;V\right)=\frac{1}{1+D}.$$

### 3.4. Proposed Model

In general, computer vision-based RSs are based upon the assumption that a user submits or picks an image of a product, and the user is then provided with similar products/images [25]. The proposed method slightly deviates from this assumption as it extracts features from past COVID-19 patients’ chest X-ray images and recommends some metadata information related to treatment alternatives based on these images.

**Figure 8:**
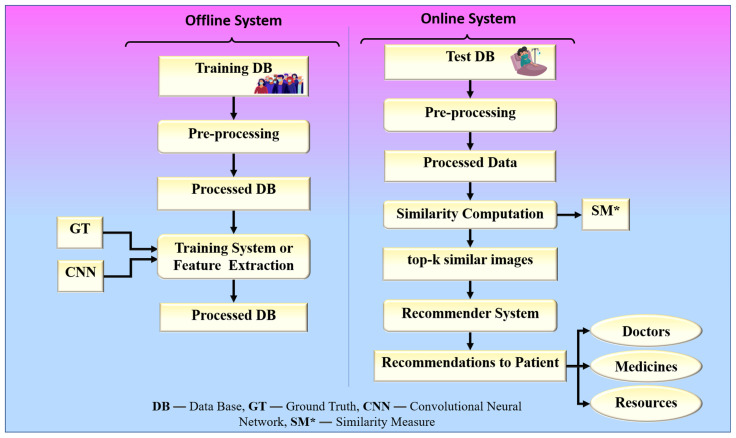
The architecture or local system for the proposed system.

The proposed framework is aimed at providing emergency solutions to hospitals during the COVID-19 pandemic using the information of past COVID-19 patients who have successfully recovered from the hospital. Therefore, it also assumes that the hospital database used to implement the proposed RS contains the chest X-ray images of COVID-19 patients who have recovered from the hospital along with the metadata (associated information) such as doctors who have investigated the patient, medicine, and resources (ICU, oxygen mask, and ventilator) provided to the patient. The architecture or the local system for the proposed RS is shown in Figure 8 and consists of two major phases: fine-tuning CNN models for feature learning (Phase-1) and recommendation (Phase-2). Phase 1 of the system is an offline system, while phase 2 is an online system.

The overall algorithm of the proposed framework is also provided in Algorithm 1. The algorithm illustrates the basic workflow of the proposed system. The system takes the chest X-ray image of a new patient as the input and recommends doctors, medicine, and hospital resources as the output. It uses a CNN model to extract the feature vectors of the input chest X-ray image and all the chest X-ray images of past COVID-19 patients stored in the hospital database. It uses a similarity measure to compute the most similar COVID-19 patients to a new patient and utilizes the metadata associated with them for the recommendations, which is represented in the testing protocol from step 5 to step 11 in the algorithm. The pre-processing and the training of the chest X-ray images are explained in the training protocol with steps from 1 to 4 in the algorithm.
**Algorithm 1:** The Overall Algorithm of the Proposed System*Training Protocol for Feature Extraction using deep learning*Obtain the chest X-ray images of COVID-19 as training data.Crop those chest X-ray images at random to 224 × 224 and rotate them at random by 30°.Input the transformed chest X-ray images obtained in step 2 into the CNN classifier for fine-tuning and begin the training of the model.When training is completed, extract the desired output layer features and save the model.*Testing Protocol using Similarity Measure*5.Obtain the chest X-ray images from the database of previous COVID-19 cases.6.Resize the chest X-ray images from the COVID-19 database to 225 × 225 and perform a centre crop of 224 × 224.7.Extract and store the feature vectors of chest X-ray images from the database using the pre-trained CNN model.8.Calculate the similarity of the query image feature vector with all the stored database feature vectors.9.Find the top-k similar feature vectors in the database, where k is a positive integer.10.Retrieve the chest X-ray images with their records of meta-data from the database, corresponding to the top-k similar feature vectors obtained in step 6.11.Recommend the doctors, medicines, and resources present in the retrieved meta-data records to the new patient as the output.

(**A**) **Phase 1 (Offline System): Fine Tuning for Feature Learning**

**Figure 9:**
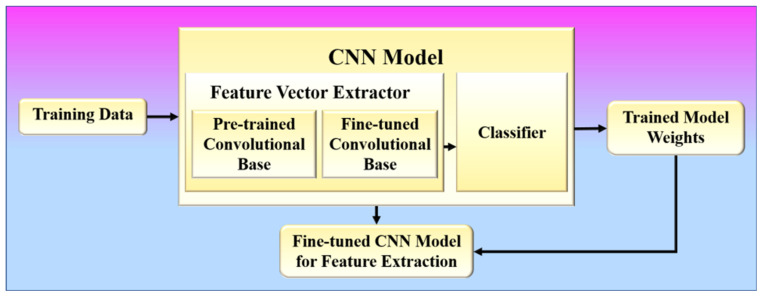
Phase-1 of the proposed system.

In phase 1, the proposed method learns to extract infection features from COVID-19 patients’ chest X-ray images based on image characteristics. A CNN model is trained to learn these features by classifying these chest X-ray images into respective lung condition categories (one of which should be COVID-19) as present in the training data. The architecture of a CNN model consists of two components: (1) feature vector extractor and (2) classifier [24,76], as shown in Figure 9.

Several convolution layers are followed by max pooling and an activation function in the feature extraction process. Typically, the classifier is made up of fully connected layers. The proposed approach uses a fine-tuning method, which is more commonly used in radiology research. It involves not only replacing the pre-trained model’s fully connected layers with a fresh set to retrain them on the given dataset but also using backpropagation to fine-tune some of the layers in the pretrained convolutional base. The binary cross-entropy loss function was used for optimization in the training of the CNN models [67,77]. The binary cross entropy loss can be defined by the following equation:

(13) $$\mathrm{Loss}=-\frac{1}{N}\sum_{i=1}^{N}y_{i.}\log\left(p\left(y_{i.}\right)\right)+\left(1-y_{i.}\right).\log\left(1-p\left(y_{i.}\right)\right).$$

During training, the *ReLU* activation function and its variations are also used because they can solve the problem of vanishing gradients, which often happens in CNN models. Before training the model for feature learning, the suggested method utilizes specific image transformations or augmentation, as shown in phase 1 [78,79]. This allows the model to be more adaptable to the huge variation in the region of interest (lungs) within the image, with less emphasis on its location, orientation, and size. Models that are trained with data transformations are more likely to improve CNN’s performance on image datasets and make them more general. In this phase, any efficient CNN model, such as VGG or ResNet, may be trained. The trained model weights are then saved, and the fine-tuned convolutional base is then employed in phase 2 to extract features. Steps 1 to 4 of the proposed algorithm shown in Figure 9 describe phase 1.

(**B**) **Phase 2 (Online System): Recommendation**

Phase 2 of the proposed approach is used for providing recommendations based on the features obtained from X-ray images using the fine-tuned convolutional base from phase 1, which acts as a feature extractor in phase 2. The metadata associated with each image in the database is utilized to provide recommendations such as doctors, medicines, and resources. For recommendation, the system utilizes similar patients from the database who have the same type of infection in the chest due to COVID-19 as that of the patient corresponding to the input query chest X-ray image. In doctor recommendation, it recommends doctors who have already successfully treated similar patients to the patient corresponding to the input query chest X-ray image. In medicine recommendation, the system recommends medicines that have already been consumed by previously recovered patients who had similar chest infections. For resource recommendations, it recommends emergency resources such as oxygen masks, ventilators, and ICU if required by the patient in the future so that the hospital can arrange those resources beforehand. Phase 2 of the proposed method is again divided into two sub-phases: (1) feature vector extraction and (2) similarity-based retrieval.

#### 3.4.1. Feature Vector Extraction

The elements or patterns of an object in an image that assist in identifying it are called “features.” Feature extraction is a step in the dimensionality reduction process, which divides and reduces a large collection of raw data into smaller groupings. It aids in extracting the most useful information from higher-dimensional data by choosing and merging variables into features, hence minimizing the amount of data. These features are easy to use and describe real data collection uniquely and accurately.

CNNs excel in extracting complex features in the form of feature vectors that depict the image in great detail, learning task-specific features while being extremely efficient [80]. Therefore, the proposed method uses CNN-based feature extractors obtained from phase 1 to extract features of the infection present inside the chest X-ray images of COVID-19 patients.

Feature vector extraction is applied both to the input query image and the chest X-ray images of COVID-19 patients present in the hospital database. Steps 5 to 10 of the proposed algorithm describe the feature vector extraction process. However, the extracted feature vectors are further exploited for similarity-based retrieval.

#### 3.4.2. Similarity-Based Retrieval

**Figure 10:**
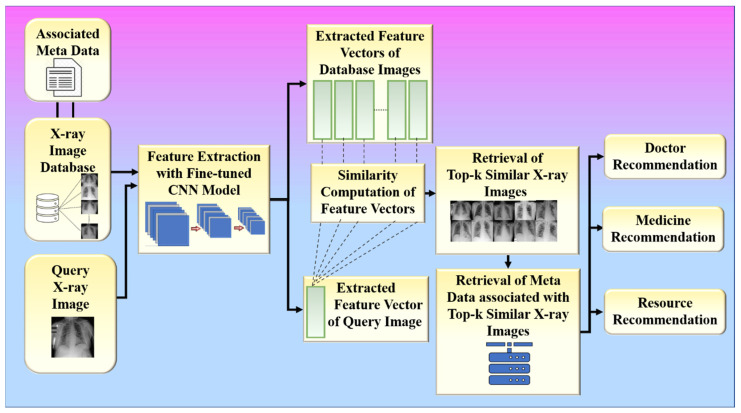
The data flow of the proposed system.

The extracted feature vectors of the input query image and the chest X-ray images of recovered COVID-19 patients present in the database obtained from the previous step are further utilized to retrieve similar images for a given input query image. The system utilizes the cosine similarity measure to find the top-k similar patients for a given query patient, where k is a positive integer. The system further utilizes those top-k similar patients to provide various recommendations such as doctors, medicines, and resources to the patient corresponding to the given input chest X-ray image. The doctors, medicines, and resources allotted to those similar patients are recommended to the query patient. Steps 11 to 14 summarize the workflow of the proposed system (Figure 10). In the results section, we present the results for the two hypotheses of our proposed system.

## 4. Experimental Protocol

To verify the efficacy of the proposed approach, the experimental environment, dataset description, pre-processing of the datasets, and the related results of the experiments are discussed in this section.

### 4.1. Experimental Environment

The details of the computing resources used for the implementation of the proposed system are shown in Table 1.

### 4.2. Dataset Description

We employed two datasets, including the chest X-ray images of COVID-19 patients, for the implementation and performance evaluation of our proposed model. The detailed descriptions of the datasets are provided in Table 2. The “Dataset for Training and Verification (DFTV)” was used for to train the CNN models and was also used for the analysis of the model’s performance. It was split into training, validation, and test sets using the K5 protocol in the ratio of 8:1:1 before training the models. In total, 16,932 images are used for training, and 2116 images are used for testing. For the performance analysis of our proposed recommendation model, all the images of the COVID class in this dataset were also taken again separately and split into five different subsets of equal size, namely DFTV-1, DFTV-2, DFTV-3, DFTV-4, and DFTV-5. The second dataset, “Dataset for Cross Verification (DFCV)”, was used as a dataset for cross-verification of the system’s performance on completely new data unseen by the CNN models. It was also split into five subsets of equal size, namely DFCV-1, DFCV-2, DFCV-3, DFCV-4, and DFCV-5.

### 4.3. Data Pre-Processing

In phase 1 of the proposed approach, the images underwent certain image transformations, as mentioned in Step 2 of the algorithm. First, the images were randomly cropped and resized to 224 × 224 and then randomly rotated by 30 degrees before going into the CNN model for training. In phase 2 of the approach, the query and the database images were pre-processed before feature extraction took place. The chest X-ray images were first resized to 225 × 225 and then cropped to size 224 × 224, facilitating the input to ResNet and VGG architectures.

## 5. Results and Performance Evaluation

The results of the proposed system were compartmentalized based on the system’s two phases. The performance of offline (Phase-1) and online (Phase-2) systems was assessed using different CNN models and similarity measures.

### 5.1. Results

The results were obtained for the two phases of the proposed system. In phase 1, the results were determined by fine-tuning the CNN model. In phase 2, the results were recorded and obtained from the recommendation process. The results were obtained by considering both the DFTV and DFCV datasets.

Phase 1: Fine Tuning for Feature Learning—Offline System

**Figure 11:**
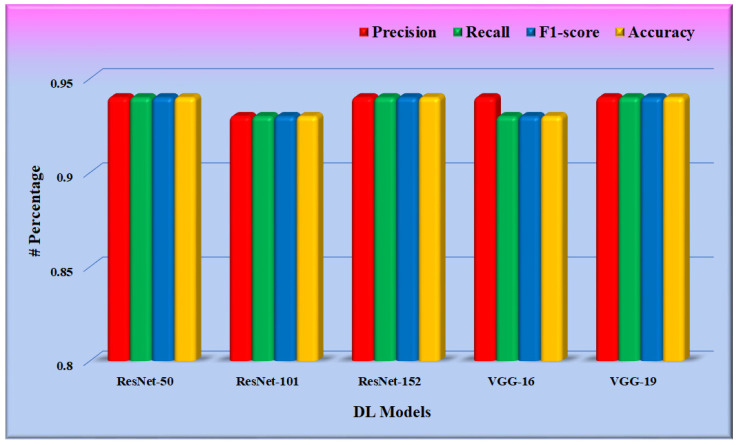
Performance evaluation of the fine-tuned CNN models.

In phase 1 of the proposed system, the CNN model was fine-tuned, and the model was saved for further use in phase 2. Training was optimized using the stochastic gradient descent (SGD) optimizer through a binary cross-entropy loss function on the DFTV dataset. Figure 11 depicts the result of these fine-tuned CNN models, as found in the test split of the dataset DFTV. The metrics used in the results are defined in the following equations.

(14) $$\mathrm{Precision}=\frac{TP\;}{TP+FP},$$

(15) $$Recall=\frac{\;TP}{\;TP+FN},$$

(16) $$F1-score=2\left.\left(\;\frac{Precision*Recall\;}{Precision+Recall}\right.\right),$$

(17) $$Accuracy=\frac{TP+TN}{Size\;of\;the\;dataset},$$

where,

*TP* is the true positive, and this is when the model correctly predicts the positive class.
*TN* is the true negative, and this is when the model correctly predicts the negative class.
*FP* is the false positive, and this is when the model incorrectly predicts the positive class.
*FN* is the false negative, and this is when the model incorrectly predicts the negative class.

From Figure 11, it is found that the weighted precision, recall, and f1-score of all the CNN models are between 0.90 and 0.95. The average weighted precision, recall, and f1-score of all five CNN models are 0.938, 0.936, and 0.936, respectively.

Phase 2: Recommendation—Online System

The final weights of the fine-tuned CNN model obtained from phase 1 were used in phase 2 for feature extraction. The weights were used for the feature extraction of both the query image and the database images. The similarity of the feature vector corresponding to the input query image was determined concerning all the feature vectors corresponding to the database images. The four similarity measures, namely, cosine similarity, Euclidean distance similarity, Jaccard similarity, and Maxwell–Boltzmann similarity, were considered for evaluating the performance of the proposed system. The results were obtained by taking all the images of the COVID-19 class present in each dataset. For each dataset, 80% of these images were considered hospital database images, i.e., chest X-ray images of past recovered COVID-19 patients of the hospital. The feature vectors were already extracted and stored in the backend, and the remaining 20% of images were considered new input query images, i.e., chest X-ray images of new patients. For each query image, the similarity with every database image was calculated, and the top-k images from the hospital database having the highest similarity were retrieved, where k is the number of recommendations. A” threshold value (T)” of similarity was decided to identify relevant similar images for each query image. A retrieved database image was considered relevant when it had a similarity greater than or equal to the threshold value, as defined in Equation (18).

*Relevant recommendation* = *retrieved database image with cosine similarity ≥ T*. (18)

We varied this threshold value between 0.7 and 0.95 to analyze different scenarios. This threshold value represents the minimum similarity of image features in the chest X-ray needed for the recommended medicines, doctors, and resources to be considered valid. This threshold value may be fixed after consulting a medical professional for practical use.

For the input query set, the average of the highest similarity corresponding to the most similar image (top-1) concerning each query was calculated and was referred to as the average highest similarity (AHS) of our proposed method, as shown in Appendix A. Table A1 and Table A2 depict the average highest similarity as observed on various datasets using different similarity measures and CNN models for feature extraction.

**Figure 12:**
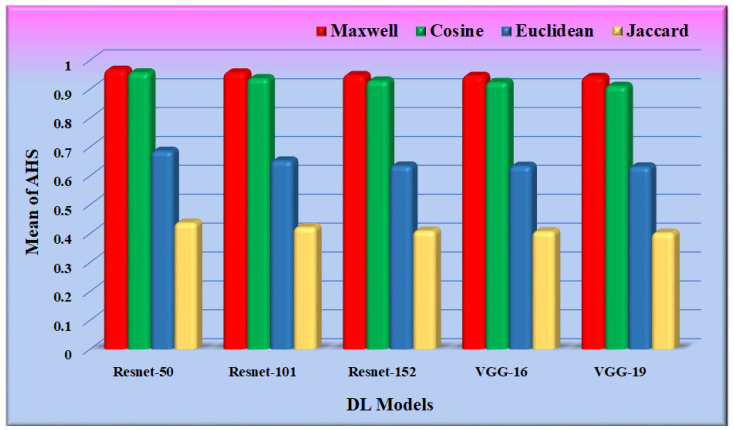
Graph for mean/standard deviation of AHS.

**Figure 13:**
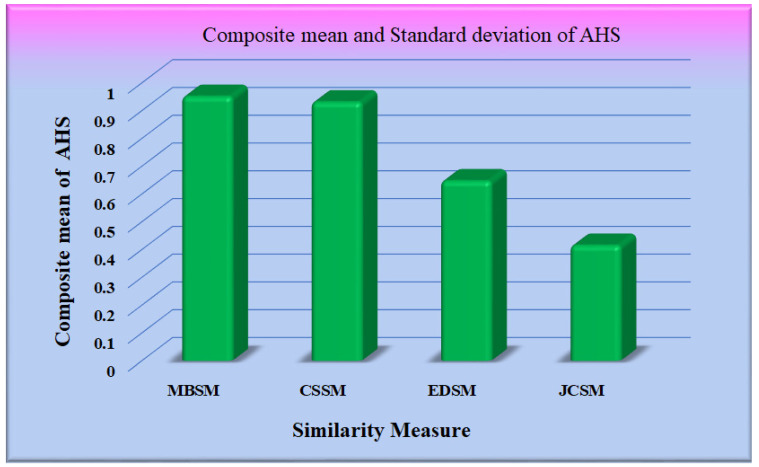
Graph for a mean of AHS.

The performance of the different similarity measures can be analyzed from the graphs shown in Figure 12. From Figure 12, it is observed that the mean of AHS of all the datasets for Maxwell–Boltzmann similarity is maximum. The performance of the Cosine similarity measure is nearer to Maxwell–Boltzmann similarity. The composite means and standard deviation of AHS considering all the datasets for all the models are represented in Figure 13. The performance of the different CNN models was further analyzed, considering Maxwell–Boltzmann similarity.

The mean average precision (*MAP@k*) metric was used to evaluate the performance of phase 2 (online system) of the proposed RS, which is defined in the following Equation (19).

(19) $$MAP@k=\frac{1}{N}\sum_{n=1}^{N}\frac{1}{k}\sum_{m=1}^{n}\left(P\left(m\right)\times rel\left(m\right)\right),$$

where *N* denotes the total number of users or the length of the input query set, *k* denotes the number of recommendations made by the recommender system, and *P*(*m*) denotes the precision up to the first m recommendations. *rel*(*m*) is a relevance indicator function for each recommended item, returning 1 if the *m*th item is a relevant recommended chest X-ray image, with a similarity higher than the threshold value *T*, and 0 otherwise. To check the performance of our proposed RS, we determined the *MAP@k* for *k* = 5 and *k* = 10. The values obtained for *MAP@k* for *k* = 5 and *k* = 10 using the five CNN feature extraction models are listed in Appendix A and are shown in Table A3 and Table A4, respectively.

**Figure 14:**
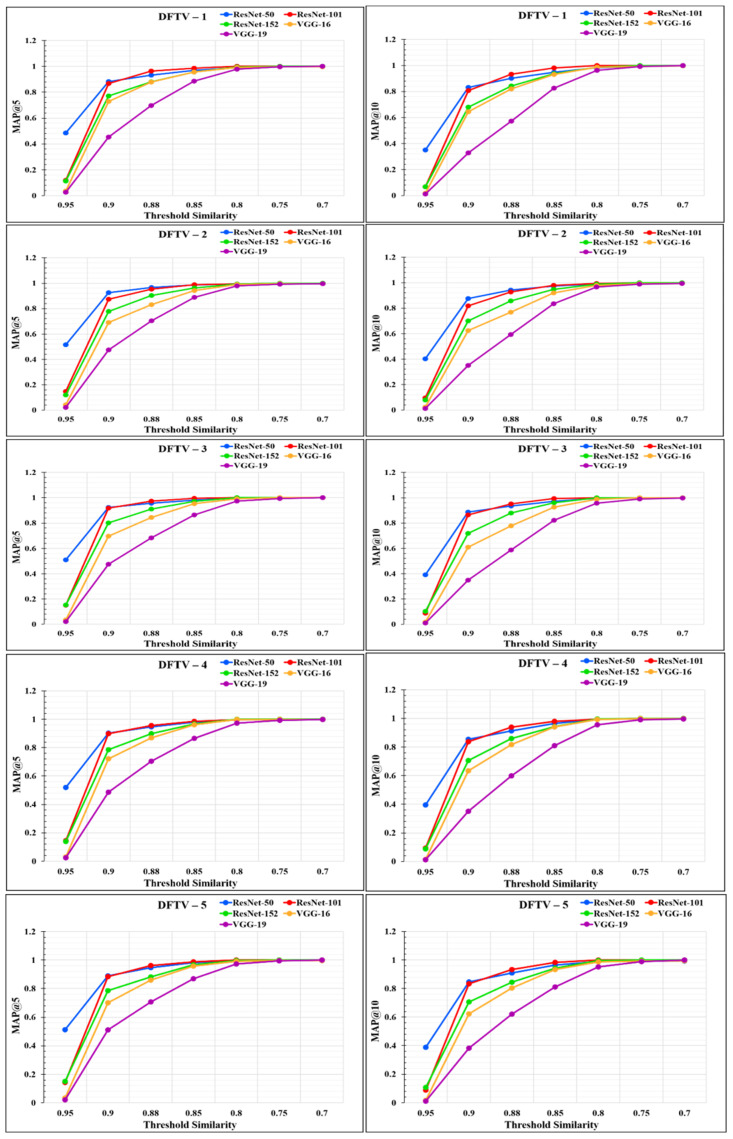
Mean average precision *@k* graphs for DFTV datasets for *k* = 5 and *k* = 10.

**Figure 15:**
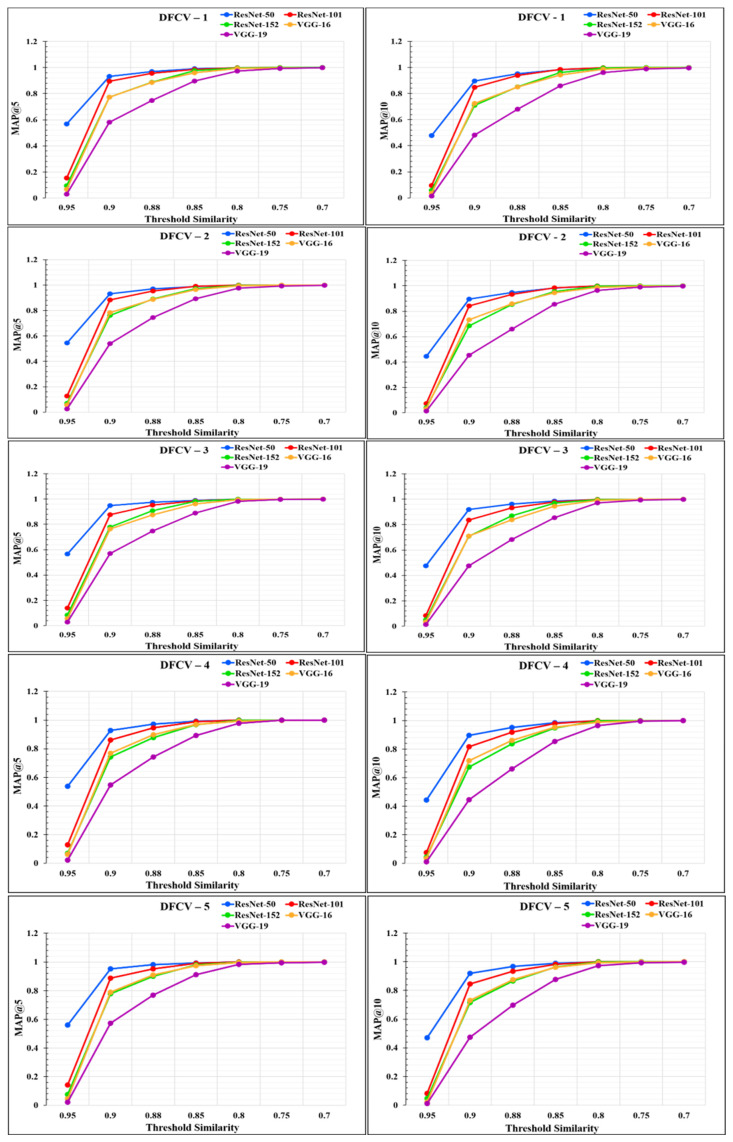
Mean Average precision @*k* graphs for DFCV datasets for *k* = 5 and *k* = 10.

The performance of the models was analyzed through the graphs represented in Figure 14 and Figure 15. From the graphs, it was observed that the performance of the proposed RS varies according to the different feature extraction methods through the different CNN models. The proposed RS implemented with the ResNet-50 feature extraction model provided the highest *MAP@k* with *k* = 5 and *k* = 10 for all the datasets with higher threshold values of similarity. The proposed RS with the ResNet-50 feature extraction CNN model had the highest MAP of more than 0.90 for the threshold similarities in the range of 0.7 to 0.9. Therefore, it confirmed the first part of the hypothesis that the performance of the proposed RS depends upon the feature extraction technique through CNN models. It was also found that this framework provides better performance for the DFCV, which follows the performance obtained from DFTV.

To analyze the effect of the similarity measures on the performance of the proposed system, we found the *MAP@*5 for both the DFTV and DFCV datasets using the Resnet-50 CNN model. We used the Resnet-50 CNN model as it is the best-performing model for our datasets. The results obtained are shown in Table 3. It was also observed from Table 3 that the *MAP@*5 was at its maximum using the Maxwell–Boltzmann similarity. Hence, the *MAP@k* depends upon the similarity measure used for similarity computation, which reveals another part of the hypothesis of our proposed system. We also validated our two hypotheses in the performance evaluation section.

### 5.2. Performance Evaluation

**Figure 16:**
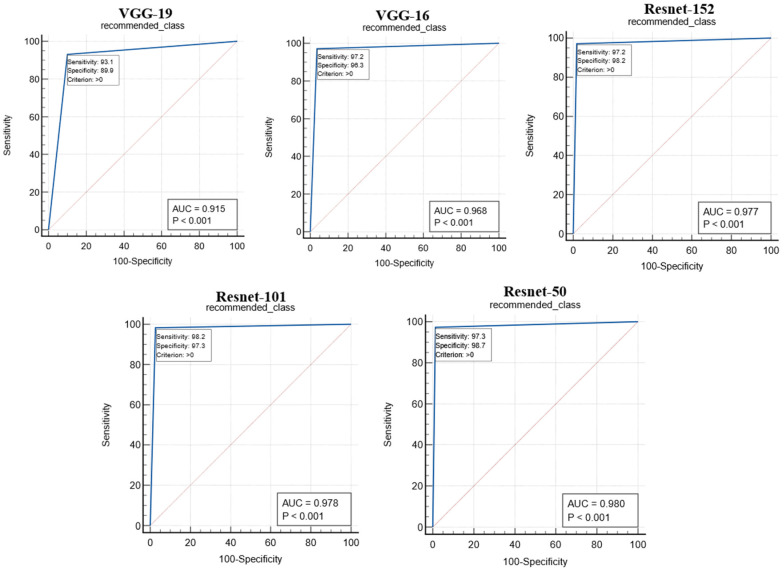
ROC curves for the performance analysis of CNN models.

The proposed study used two performance metrics, (i) the ROC curve and (ii) the figure of merit (*FoM*), to validate the performance of the proposed system. The ROC curves for the performance of the different CNN models are shown in Figure 16. The ROC curve represents the ability of the CNN models in feature extraction so that the predicted value of the recommended image matches the gold standard. The performance of the CNN models was analyzed with the area under the curve (AUC) and the corresponding *p*-value, as shown in Table 4. The Resnet-50 model was found to outperform other CNN models with an AUC.

The performance of the CNN models was also analyzed through *FoM*. The *FoM* is defined as the error’s central tendency and can be defined as follows:

(20) $$FoM=100-\left[\left(\frac{W}{N}\right)\times 100\right],$$

where *W* is the number of images incorrectly classified according to the GT, and *N* is the total number of images present in the test sample. Table 5 displays the *FoM* values for the proposed RS with different CNN models and Maxwell–Boltzmann similarity measures in comparison to the GT. Hence, the two results of the ROC curve and *FoM* values found in Table 4 and Table 5 validate that the performance of the proposed RS varies according to the different CNN models used for feature extraction. This, in turn, validates the first part of our hypothesis.

We also determined the *FoM* values considering the four similarity measures keeping the CNN model fixed. We considered Resnet-50 as the best-performing CNN model observed from the previous results. The *FoM* values obtained using Resnet-50 and the four similarity measures are shown in Table 6. It was found that the *FoM* was at its maximum for Maxwell–Boltzmann similarity and varied according to the similarity measure used in the system. This result validates the second part of our hypothesis that the performance of the proposed RS depends upon the similarity measure used for similarity computation.

### 5.3. Running Time Comparison

**Figure 17:**
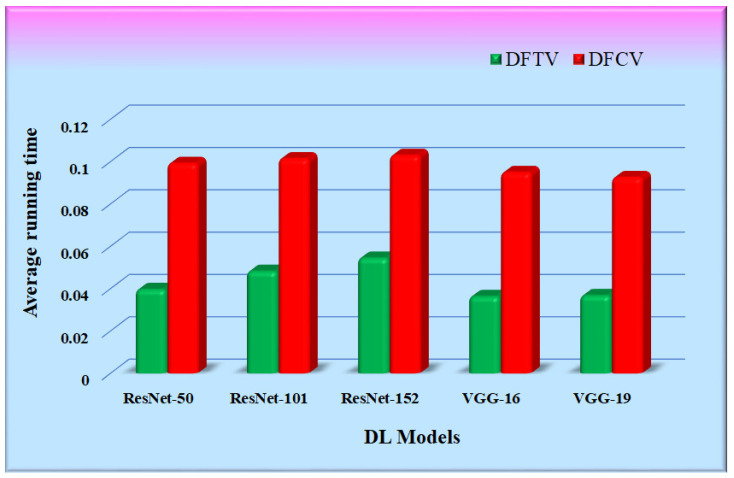
Graphs for average running time comparison.

Table 7 shows the time consumed by the proposed RS with each of the five CNN feature extraction algorithms. It is the average of multiple runs that have been expressed in seconds. The working setup to conduct experimentation is shown in Table 1. The running time of the proposed RS was calculated as the time required for the feature extraction of an input query image supplied to the proposed RS, its similarity calculation with all the images in the hospital database, and the retrieval of top-k similar images. The average running time of the proposed RS implemented with the CNN feature extraction models was compared and is represented in Figure 17. The bars in the figure represent the average running time of each RS with different CNN models. It was observed that the running time of the proposed RS is primarily dependent upon the size of the hospital database and is also affected by the type of feature extraction model.

### 5.4. Statistical Tests

The proposed study performed the validations of the two hypotheses designed for the proposed system. To assess the system’s reliability and stability, the standard Mann–Whitney, paired *t*-test, and Wilcoxon tests were used. When the distribution was not normal, the Wilcoxon test was used instead of the paired *t*-test to determine whether there was sufficient evidence to support the hypothesis. MedCalc software (Osteen, Belgium) was used for the statistical analysis. To validate the system proposed in the study, we provided all of the *MAP@k* values for *k* = 5 and *k* = 10 against various models of RS with different CNNs. The results of the Mann–Whitney, paired *t*-test, and Wilcoxon test are shown in Table 8.

## 6. Discussion

### 6.1. Principal Findings

**Figure 18:**
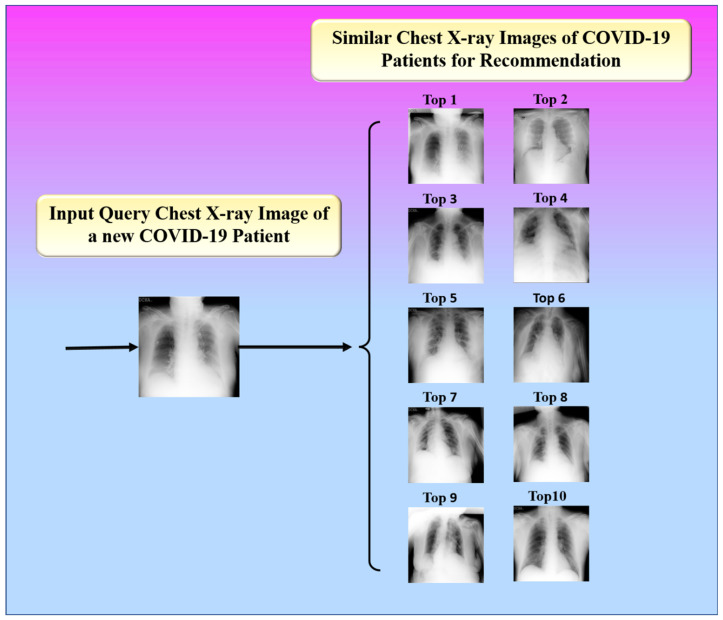
Top 10 similar images for recommendation.

The proposed study presented an image-based health RS for the efficient management of resources in hospitals such as doctors, medicine, ICUs, ventilators, and oxygen masks during the peak period of the COVID-19 pandemic. The proposed system recommends these resources to a new patient according to his or her current health condition. It is defined as a hybrid of an offline and an online system. The offline system is in charge of extracting feature vectors from images. Using a similarity measure, the online system compares the feature vectors of the image being queried and the image in the database. The top-k most similar images are then found, as shown in Figure 18.

The test was carried out on 20,000 COVID-19 patients’ chest X-ray images. The following similarity measures were used to select the best one for the system based on the AHS value: (i) cosine similarity, (ii) Maxwell–Boltzmann similarity, (iii) Euclidean similarity, and (iv) Jaccard similarity. With a similarity value of more than 94%, the Maxwell–Boltzmann similarity outperformed all other similarity measures. The proposed RS’ performance was validated using the following CNN models: (i) Resnet-50, (ii) Resnet-101, (iii) Resnet-152, (iv) VGG-16, and (v) VGG-19. The performance of the CNN models was validated using parameters such as the ROC curve and *FoM* value. The AUC and *p*-values obtained from the ROC curve indicate the ability of the CNN models to correctly predict the GT of the input image. The Resnet-50 model was found to outperform other CNN models with an AUC greater than 0.98 (*p* < 0.0001). The performance of the CNN models was also analyzed through *FoM*. The *FoM* was defined as the error’s central tendency. The Resnet-50 CNN model was found to have a maximum *FoM* value of 98.38. The performance of the similarity measures was also validated using the *FoM* value, and Maxwell–Boltzmann similarity outperformed the other three similarity measures; the overall performance of the proposed RS was evaluated using *MAP@k*. The *MAP@k* was determined using different CNN models for the threshold similarity in the range of 0.7 to 0.95. The proposed RS with the Resnet-50 CNN model showed the best result with a *MAP@k* value of 0.98014 and 0.98861 for *k* = 5 and *k* = 10, respectively. Finally, the system recommended meta-data information regarding hospital resources to a new COVID-19 patient admitted to the hospital based on his or her chest X-ray image. 

### 6.2. Benchmarking

We considered various papers related to RS based on image similarity in our benchmarking strategy. This included Ullah et al. [17], Chen et al. [18], Tuinhof et al. [19], and Geng et al. [40]. In ref. [17], an RS based on image content was proposed and divided into two phases. The RS used a random forest classifier in the first phase to determine the product’s class or category. The system then used the JPEG coefficients measure to extract the feature vectors of the photos, which were then used to provide recommendations based on feature vector similarity in the second phase. The proposed method produced correct recommendations with a 98% accuracy rate, indicating its efficacy in real-world applications. Ref. [18] provided a neural network-based framework for product selection based on a specific input query image. A neural network was used in the proposed system to classify the supplied input query image, followed by another neural network that used the Jaccard similarity measure to determine the most comparable product image to that input image. The approach had a classification accuracy of 0.5. It offered quick and accurate online purchasing assistance and recommended products with a similarity of more than 0.5. Ref. [19] describes a two-stage deep learning framework for recommending fashion images based on similar input images. The authors proposed using a neural network classifier as a data-driven, visually aware feature extractor. The data were then fed into ranking algorithms, which generated suggestions based on similarities. The proposed method was validated using the fashion dataset, which was made public. The proposed framework, when combined with other types of content-based recommendation systems, can improve the system’s stability and effectiveness. Ref. [40] proposed a framework for combining an RS with visual product attributes by employing a deep architecture and a series of convolution operations that result in the overlapping of edges and blobs in images. The benchmarking table for the proposed study is shown in Table 9.

The proposed framework for developing an entirely image-based recommendation model compares various linear and nonlinear reduction approaches to the properties of a CNN. Ref. [82] presented an RS framework that uses chest X-ray images to predict whether a person needs COVID-19 testing. It implemented the same datasets used by the proposed method but with a different objective. None of these studies proposed any hypothesis for their proposed systems.

In contrast, we proposed two hypotheses for our system and also evaluated and validated them in the result and performance evaluation sections, respectively.

### 6.3. Special Note on Searching for RS

RS works on the principle of information filtering, and the searching strategy plays an important role in finding the relevant items to produce efficient and useful recommendations. The proposed RS utilizes image similarity to find the most relevant chest X-ray images with similar infections for a new COVID-19 patient with a chest X-ray image. Although CNN models play a vital role in producing accurate feature vectors, the quality of the recommendation mainly depends on the similarity measure. A proper similarity measure producing a high similarity value can produce more accurate recommendations. The four similarity measures considered for this study were analyzed based on AHS. In this study, the AHS was determined by averaging the similarity value of the most similar image to every input image present in the test set. The similarity measure with the highest AHS was considered for the RS. The performance of the proposed RS was determined in terms of *MAP@k* for a top-k recommendation. To identify relevant similar images for each query image, a “threshold value (T)” of similarity was also considered in the system. A retrieved database image was considered relevant when it had a similarity greater than or equal to the threshold value. This threshold value was found to affect the overall performance of the system in terms of *MAP@k* for a top-k recommendation.

The input images in both the training set and the testing sets were large images. These large images had many pixels to process. Further, the method we adopted reduced the computational complexity. The similarity measure strategy was very fast, quick, and low in complexity, one reason being there was no special optimization protocol and iteration adopted. Thus, overall, there was simplicity, speed, and low complexity. Such benefits overrule direct image comparison. Note that the top-n similar images obtained from the similarity computation were used for the recommendation. The proposed RS using CNN for feature extraction and similarity measurement can be an efficient tool to produce recommendations in the healthcare domain. The recommendations can be utilized for the proper allocation of doctors, medicine, and hospital resources to new patients.

### 6.4. Strengths, Weaknesses, and Extensions

The proposed method shows that the RS using a CNN for feature extraction and similarity measure can be an efficient tool for producing recommendations in the healthcare domain. The recommendations can be utilized for the proper allocation of doctors, medicine, and hospital resources to new patients. The proposed study proposed two hypotheses and also evaluated and validated them in the paper.

The results of the current pilot study are encouraging. However, due to the unavailability of the denoising technique in the proposed RS, the quality of the recommendation may be affected due to the presence of noise in the chest X-ray images. Denoising can be conducted in the offline and online systems. Denoising is an expensive operation in terms of computations. Therefore, offline denoising does not hurt the system that much, but the online system must be hardware interactive. The low resolution of chest X-ray images may also affect the quality of recommendations. Due to the limited number of images available for similarity calculation, a small database size may result in incorrect recommendations. A large database size may result in longer training time. While the study used basic ResNet-based systems, this can be extended to hybrid ResNet systems [83,84].

In the future, we could apply more sophisticated feature extraction techniques by fusing the different deep-learning models to achieve accurate recommendations. Better similarity methods can be explored to increase the efficiency of the proposed system. It could also be enhanced by applying segmentation techniques to make the system more robust. It can also be extended to cloud settings and big data platforms.

## 7. Conclusions

Through this study, we offered an RS for treating COVID-19 patients based on X-ray images of the chest. The proposed RS was divided into two phases. In phase 1, the proposed system fine-tuned the CNN models for feature extraction in phase 2. In phase 2, the finely tuned CNN model was used to extract features from both the chest X-ray of a new COVID-19 patient and the chest X-rays of COVID-19 patients present in the hospital database who were already treated successfully. The top-k similar images to the input query image of a new COVID-19 patient were determined further utilized for recommendation. In its recommendation, the proposed RS recommends doctors, medicines, and resources for new COVID-19 patients according to the metadata information of similar patients.

The proposed RS implemented with the ResNet-50 feature extraction CNN model provides the highest *MAP@k* with *k* = 5 (top-5) and *k* = 10 (top-10) for all the datasets with higher threshold values of similarity. The proposed RS with ResNet-50 CNN feature extraction model was found to be a proper framework for the treatment recommendation with a mean average precision (MAP) of more than 0.90 for the threshold similarities in the range of 0.7 to 0.9. The results of the proposed study were hypothesized and validated using various parameters. The proposed RS in this paper assumes that the hospital database contains related metadata, such as information about the doctors investigated, medicines, and resources allocated to a patient. The major limitation of our proposed system is that we did not consider the related physiological parameters such as sugar level, blood pressure, and other associated parameters that may affect the condition of a COVID-19 patient having similar chest infections. In the future, the proposed RS can be enhanced by considering these parameters for better recommendations.

## Figures and Tables

**Table 1:** Computer resources.

Software and Hardware	Specifications
Operating System	Debian GNU/Linux 9 (stretch)
CPU	Intel(R) Xeon(R) CPU @ 2.00GHz
GPU	GPU—Tesla P100 16GB
Language	Python 3.9
RAM	16 GB
Disk	645 GB

**Table 2:** Description of datasets.

	DFTV	DFCV
Dataset Name	**COVID-19 Radiography Database**	**COVID-QU-Ex Dataset**
Dataset link	Link1 [81]	Link2 [45]
Dataset size	781 MB	329 MB
Dimensions	299 × 299	256 × 256
Number of Images	21,165	11,956
Number of Classes	4	1

DFTV: Data set for Training and verification, DFCV: Data set for Cross Validation.

**Table 3:** Mean average precision @5 for DFTV and DFCV datasets using the Resnet-50 CNN model.

Datasets	EDSM	JCSM	CSSM	MBSM
**DFTV-1**	0.50127	0.62346	0.91724	0.94827
**DFTV-2**	0.52037	0.63658	0.93586	0.9731
**DFTV-3**	0.54386	0.66215	0.95034	0.97068
**DFTV-4**	0.49873	0.61384	0.92586	0.95224
**DFTV-5**	0.50012	0.61931	0.93793	0.96413
**DFCV-1**	0.53247	0.64034	0.95282	0.98328
**DFCV-2**	0.49771	0.61241	0.93517	0.98129
**DFCV-3**	0.51023	0.62011	0.94387	0.98495
**DFCV-4**	0.53387	0.63286	0.95672	0.9839
**DFCV-5**	0.53218	0.65238	0.96458	0.98861

**Table 4:** AUC and *p*-value for the CNN models.

SN	CNN Model	AUC	*p*-Value
1	VGG-19	0.915	*p* < 0.001
2	VGG-16	0.968	*p* < 0.001
3	Resnet-152	0.977	*p* < 0.001
4	Resnet-101	0.978	*p* < 0.001
5	Resnet-50	0.980	*p* < 0.001

**Table 5:** *FoM* was observed for the CNN models.

SN	CNN Model	*FoM* (%)
1	VGG-19	91.12
2	VGG-16	96.23
3	Resnet-152	97.04
4	Resnet-101	97.84
5	Resnet-50	98.38

**Table 6:** *FoM* was observed for the similarity measures.

SN	CNN Model	*FoM* (%)
1	JCSM	42.74
2	EDSM	58.39
3	CSSM	97.26
4	MBSM	98.38

**Table 7:** Average running time obtained for the CNN models.

		Average Running Time (in Seconds)			
**Datasets**	**ResNet-50**	**ResNet-101**	**ResNet-152**	**VGG-16**	**VGG-19**
**DFTV**	0.037	0.063	0.061	0.041	0.034
**DFCV**	0.098	0.101	0.103	0.094	0.092

**Table 8:** Results of statistical tests.

Models	Mann–Whitney	Paired *t*-Test	Wilcoxon Test
M1 vs. M2	*p* < 0.0001	*p* < 0.0001	*p* < 0.0001
M1 vs. M3	*p* < 0.0001	*p* < 0.0001	*p* < 0.0001
M1 vs. M4	*p* < 0.0001	*p* < 0.0001	*p* < 0.0001
M1 vs. M5	*p* < 0.0001	*p* < 0.0001	*p* < 0.0001
M2 vs. M3	*p* < 0.0001	*p* < 0.0001	*p* < 0.0001
M2 vs. M4	*p* < 0.0001	*p* < 0.0001	*p* < 0.0001
M2 vs. M5	*p* < 0.0001	*p* < 0.0001	*p* < 0.0001
M3 vs. M4	*p* < 0.0001	*p* < 0.0001	*p* < 0.0001
M3 vs. M5	*p* < 0.0001	*p* < 0.0001	*p* < 0.0001
M4 vs. M5	*p* < 0.0001	*p* < 0.0001	*p* < 0.0001

M1—RS using Resnet-50, M2—RS using Resnet-101, M3—RS using Resnet-152, M4—RS using VGG-16, M5—RS using VGG-19.

**Table 9:** Benchmarking table.

	C1	C2	C3	C4	C5	C6	C7	C8
SN	Author	Number of Images	Technique	Model Types	ACC	MAP	Loss	AHS
1	Ullah et al. [25]	2000	Feature Extraction by JPEG Coefficient and Classification by RF	Cosine	NR	NR	NR	0.967
				Euclidean	NR	NR	NR	0.961
				Subjective	NR	NR	NR	0.93
2	Chen et al. [82]	10,000	Neural Network and Jaccard Similarity	SVM	0.26790	NR	NR	NR
				Alexnet	0.39460	NR	NR	NR
				VGG	0.5010	NR	NR	NR
3	Tuinhof et al. [27]	11,851	Feature Extraction and ranking by KNN	Alexnet	NR	NR	1.48	NR
				BN-Inception	NR	NR	1.27	NR
4	Geng et al. [51]	686,457	Feature Learning	CBF	NR	0.098	NR	NR
				UCF	NR	0.308	NR	NR
				ICF	NR	0.338	NR	NR
				WMF	NR	0.356	NR	NR
				DW	NR	0.550	NR	NR
				DUIF	NR	0.457	NR	NR
5	**Kuanr et al. (proposed)**	20,000	Feature Extraction and Similarity by Maxwell–Boltzmann Similarity	VGG-19	91.12	0.85318	1.39	0.9428
				VGG-16	96.23	0.95308	1.21	0.9466
				Resnet-152	97.04	0.94806	1.26	0.9449
				Resnet-101	97.84	0.97931	1.17	0.9567
				Resnet-50	98.38	0.984375	1.09	0.9668

## Data Availability

Used data is available in Kaggle website.

## Abbreviations

**SN**	**Abb ***	**Definition**
1	AHS	Average Highest Similarity
2	AUC	Area-under-the-curve
3	AI	Artificial Intelligence
4	ARDS	Acute Respiratory Distress Syndrome
5	BMI	Body mass index
6	CSSM	Cosine Similarity Measure
7	CNN	Convolution neural network
8	EDSM	Euclidean Distance Similarity Measure
9	GGO	Ground Glass Opacities
10	VGG	Visual Geometry Group
11	DL	Deep learning
12	DM	Diabetes mellitus
13	Densenet	Dense Convolutional Network
14	MBSM	Maxwell–Boltzmann Similarity Measure
15	MAP	Mean Average Precision
16	ML	Machine Learning
17	KNN	K- nearest neighbor
18	*FoM*	Figure of Merit
19	HDL	Hybrid deep learning
20	HRS	Health Recommender System
21	NB	Naive Bayes
22	NR	Not reported
23	PCA	Principal component analysis
24	PI	Pulmonary Infiltrates
25	Resnet	Residual Neural Network
26	PR	The period measured in milliseconds
27	RF	Random forest
28	ReLU	Rectified Linear Activation Unit
29	RoB	Risk of bias
30	ROC	Receiver operating-characteristics
31	TL	Transfer learning
32	DFTV	Dataset for Training and Verification
33	DFCV	Dataset for Training and Cross Validation

## Appendix A

**Table A1:** Average highest cosine similarity (AHS) obtained for the CNN models with DFTV dataset.

Dataset	Similarity Measure	Feature Vector Extractor Architecture				
		ResNet-50	ResNet-101	ResNet-152	VGG-16	VGG-19
**DFTV-1**	CSSM	0.9557	0.9383	0.9298	0.9233	0.9109
	EDSM	0.6834	0.6517	0.6326	0.6338	0.6319
	JCSM	0.4369	0.4210	0.4098	0.4049	0.4016
	MBSM	0.9651	0.9588	0.9489	0.9457	0.9421
**DFTV-2**	CSSM	0.9587	0.9376	0.9300	0.9202	0.9090
	EDSM	0.6858	0.6511	0.6374	0.6304	0.6308
	JCSM	0.4394	0.4207	0.4117	0.4125	0.4039
	MBSM	0.9676	0.9564	0.9510	0.9428	0.9392
**DFTV-3**	CSSM	0.9572	0.9392	0.9327	0.9216	0.9089
	EDSM	0.6850	0.6536	0.6388	0.6321	0.6306
	JCSM	0.4383	0.4228	0.4120	0.4107	0.4041
	MBSM	0.9668	0.9579	0.9533	0.9445	0.9385
**DFTV-4**	CSSM	0.9566	0.9381	0.9324	0.9244	0.9114
	EDSM	0.6859	0.6541	0.6376	0.6346	0.6328
	JCSM	0.4376	0.4234	0.4122	0.4086	0.4066
	MBSM	0.9670	0.9581	0.9528	0.9440	0.9419
**DFTV-5**	CSSM	0.9572	0.9385	0.9319	0.9238	0.9100
	EDSM	0.6854	0.6548	0.6380	0.6335	0.6318
	JCSM	0.4370	0.4230	0.4118	0.4027	0.4006
	MBSM	0.9665	0.9587	0.9530	0.9431	0.9416
**Mean**		0.762	0.743	0.733	0.727	0.721
**Standard Deviation**		0.224	0.227	0.229	0.227	0.225

**Table A2:** Average highest cosine similarity (AHS) obtained for the CNN models with DFCV dataset.

Dataset	Similarity Measure	Feature Vector Extractor Architecture				
		ResNet-50	ResNet-101	ResNet-152	VGG-16	VGG-19
**DFCV-1**	CSSM	0.9582	0.9379	0.9281	0.9256	0.9121
	EDSM	0.6874	0.6528	0.6332	0.6347	0.6323
	JCSM	0.4384	0.4218	0.4098	0.4069	0.4011
	MBSM	0.9677	0.9580	0.9465	0.9472	0.9437
**DFCV-2**	CSSM	0.9570	0.9359	0.9254	0.9263	0.9103
	EDSM	0.6861	0.6514	0.6326	0.6357	0.6308
	JCSM	0.4368	0.4206	0.4066	0.4074	0.4006
	MBSM	0.9663	0.9564	0.9433	0.9482	0.9418
**DFCV-3**	CSSM	0.9587	0.9363	0.9280	0.9252	0.9122
	EDSM	0.6882	0.6522	0.6335	0.6345	0.6319
	JCSM	0.4370	0.4220	0.4083	0.4054	0.4018
	MBSM	0.9670	0.9559	0.9457	0.9468	0.9435
**DFCV-4**	CSSM	0.9565	0.9351	0.9250	0.9262	0.9104
	EDSM	0.6866	0.6519	0.6317	0.6360	0.6312
	JCSM	0.4355	0.4215	0.4058	0.4068	0.4013
	MBSM	0.9657	0.9552	0.9436	0.9474	0.9415
**DFCV-5**	CSSM	0.9580	0.9367	0.9273	0.9259	0.9126
	EDSM	0.6871	0.6518	0.6329	0.6352	0.6327
	JCSM	0.4362	0.4221	0.4074	0.4074	0.4027
	MBSM	0.9668	0.9567	0.9449	0.9466	0.9428
**Mean**		0.762	0.742	0.728	0.729	0.722
**Standard Deviation**		0.225	0.226	0.228	0.229	0.227

**Table A3:** Mean average precision (MAP) @*K* = 5 of CNN model.

Datasets	Threshold Similarity (T)	ResNet-50	ResNet-101	ResNet-152	VGG-16	VGG-19
**DFTV-1**	T = 0.70	1	1	1	0.99724	1
	T = 0.75	0.99793	1	1	0.99586	0.99655
	T = 0.80	0.98965	1	0.99448	0.99034	0.97724
	T = 0.85	0.96965	0.98620	0.95655	0.95586	0.88551
	T = 0.88	0.93241	0.96275	0.87931	0.87793	0.69724
	T = 0.90	0.88275	0.86758	0.77034	0.72827	0.45379
	T = 0.95	0.48551	0.11931	0.11241	0.03517	0.02551
**DFTV-2**	T = 0.70	0.99931	1	1	0.99586	0.99586
	T = 0.75	0.99793	1	1	0.99517	0.99172
	T = 0.80	0.99379	0.99448	0.99448	0.98965	0.97862
	T = 0.85	0.98551	0.98758	0.96206	0.94275	0.88896
	T = 0.88	0.96689	0.95379	0.90344	0.83103	0.70275
	T = 0.90	0.92551	0.87379	0.77793	0.69103	0.47379
	T = 0.95	0.51517	0.14758	0.11931	0.042068	0.02137
**DFTV-3**	T = 0.70	1	1	1	1	1
	T = 0.75	0.99862	1	1	1	0.99379
	T = 0.80	0.99793	1	1	0.99241	0.97379
	T = 0.85	0.98068	0.99586	0.97103	0.95310	0.86482
	T = 0.88	0.95586	0.97241	0.90965	0.84344	0.68275
	T = 0.90	0.92413	0.91793	0.80137	0.69517	0.47379
	T = 0.95	0.50965	0.15034	0.15241	0.03379	0.02206
**DFTV-4**	T = 0.70	0.99862	1	1	0.99862	0.99862
	T = 0.75	0.99862	1	1	0.99862	0.99241
	T = 0.80	0.99724	0.99655	1	0.99793	0.97172
	T = 0.85	0.98	0.98620	0.96758	0.96137	0.86551
	T = 0.88	0.94689	0.95586	0.89793	0.86827	0.70413
	T = 0.90	0.90206	0.89931	0.78482	0.72137	0.48551
	T = 0.95	0.51862	0.14344	0.13931	0.02965	0.02482
**DFTV-5**	T = 0.70	0.99586	1	1	0.99724	1
	T = 0.75	0.99586	1	1	0.99586	0.99517
	T = 0.80	0.99586	1	0.99724	0.99172	0.97241
	T = 0.85	0.98137	0.98758	0.96758	0.95724	0.86896
	T = 0.88	0.94689	0.96137	0.88206	0.85931	0.70689
	T = 0.90	0.89034	0.88344	0.78620	0.70068	0.51034
	T = 0.95	0.51310	0.14206	0.14965	0.03448	0.02137
**DFCV-1**	T = 0.70	1	1	1	0.99874	0.99791
	T = 0.75	0.99874	1	1	0.99686	0.99289
	T = 0.80	0.99665	0.99916	0.99686	0.99247	0.97262
	T = 0.85	0.99143	0.98620	0.97617	0.95903	0.89655
	T = 0.88	0.96907	0.95611	0.88777	0.88631	0.74670
	T = 0.90	0.93187	0.89487	0.77324	0.77220	0.58098
	T = 0.95	0.56823	0.15423	0.09404	0.06645	0.02884
**DFCV-2**	T = 0.70	1	1	1	0.99979	0.99811
	T = 0.75	0.99958	1	1	0.99895	0.99247
	T = 0.80	0.99895	0.99958	0.99749	0.99331	0.97471
	T = 0.85	0.98766	0.99038	0.97115	0.96489	0.89299
	T = 0.88	0.96907	0.95276	0.88965	0.88714	0.74461
	T = 0.90	0.93207	0.88234	0.76154	0.78265	0.53939
	T = 0.95	0.54503	0.126227	0.07042	0.05642	0.02549
**DFCV-3**	T = 0.70	0.99916	0.99916	1	1	0.99937
	T = 0.75	0.99895	0.99895	0.99937	0.99937	0.99686
	T = 0.80	0.99853	0.99853	0.99832	0.99498	0.98202
	T = 0.85	0.98996	0.98537	0.98140	0.96196	0.88965
	T = 0.88	0.97471	0.95276	0.90804	0.87502	0.74775
	T = 0.90	0.94858	0.87628	0.77868	0.76426	0.56907
	T = 0.95	0.56677	0.13939	0.08296	0.05621	0.02737
**DFCV-4**	T = 0.70	1	1	1	0.99958	1
	T = 0.75	0.99895	1	1	0.99791	0.99832
	T = 0.80	0.99853	0.99916	0.99811	0.99122	0.97763
	T = 0.85	0.99247	0.98829	0.96760	0.96907	0.89153
	T = 0.88	0.97178	0.94503	0.87711	0.89822	0.74252
	T = 0.90	0.92789	0.86060	0.74169	0.76823	0.54670
	T = 0.95	0.53772	0.12936	0.07021	0.06269	0.02110
**DFCV-5**	T = 0.70	1	1	1	1	0.99811
	T = 0.75	1	1	1	0.99874	0.99477
	T = 0.80	0.99853	1	0.99811	0.99644	0.98244
	T = 0.85	0.99226	0.99017	0.97868	0.97387	0.91055
	T = 0.88	0.98014	0.95193	0.89926	0.90888	0.76781
	T = 0.90	0.95047	0.88756	0.77680	0.78871	0.57199
	T = 0.95	0.55945	0.14211	0.07439	0.04472	0.02110

**Table A4:** Mean average precision (MAP) @*K* = 10 of CNN model.

Datasets	Threshold Similarity (T)	ResNet-50	ResNet-101	ResNet-152	VGG-16	VGG-19
**DFTV-1**	T = 0.70	0.99827	1	1	0.99689	0.99965
	T = 0.75	0.99310	1	1	0.99275	0.99206
	T = 0.80	0.98275	0.99896	0.98620	0.98689	0.96206
	T = 0.85	0.94827	0.98034	0.93586	0.93103	0.82586
	T = 0.88	0.90206	0.93344	0.84137	0.81965	0.57241
	T = 0.90	0.83172	0.80793	0.67896	0.64517	0.32862
	T = 0.95	0.35103	0.06827	0.06724	0.01758	0.01275
**DFTV-2**	T = 0.70	0.99655	1	1	0.99344	0.99448
	T = 0.75	0.99551	0.99827	1	0.99103	0.98724
	T = 0.80	0.98965	0.99379	0.98862	0.97965	0.96586
	T = 0.85	0.97310	0.97827	0.94758	0.91931	0.83413
	T = 0.88	0.94137	0.92620	0.85689	0.76793	0.59172
	T = 0.90	0.87448	0.81758	0.69931	0.62379	0.35103
	T = 0.95	0.40275	0.09517	0.07931	0.02275	0.01137
**DFTV-3**	T = 0.70	1	1	1	1	0.99724
	T = 0.75	0.99758	1	1	1	0.98931
	T = 0.80	0.99620	1	0.99862	0.98862	0.95655
	T = 0.85	0.97068	0.99379	0.96	0.92517	0.82206
	T = 0.88	0.93551	0.95172	0.87965	0.77827	0.58724
	T = 0.90	0.88586	0.86482	0.71758	0.60896	0.34931
	T = 0.95	0.39172	0.08758	0.10310	0.01689	0.01103
**DFTV-4**	T = 0.70	0.99758	1	1	0.99758	0.99586
	T = 0.75	0.99689	1	1	0.99758	0.99034
	T = 0.80	0.99344	0.99448	0.99551	0.99310	0.95586
	T = 0.85	0.96379	0.97862	0.94275	0.94103	0.80931
	T = 0.88	0.91241	0.93827	0.85862	0.81586	0.59827
	T = 0.90	0.85344	0.83620	0.70655	0.63310	0.35172
	T = 0.95	0.39482	0.09344	0.08862	0.01482	0.01241
**DFTV-5**	T = 0.70	0.99275	1	1	0.99344	0.99896
	T = 0.75	0.99275	1	1	0.99275	0.98862
	T = 0.80	0.98965	0.99896	0.99655	0.98758	0.95206
	T = 0.85	0.96413	0.98379	0.94310	0.93344	0.81103
	T = 0.88	0.90896	0.93379	0.84448	0.80379	0.62068
	T = 0.90	0.84620	0.83413	0.70620	0.62310	0.38310
	T = 0.95	0.38793	0.08793	0.10620	0.01724	0.01068
**DFCV-1**	T = 0.70	0.99947	1	1	0.99791	0.99655
	T = 0.75	0.99759	1	1	0.99571	0.98819
	T = 0.80	0.99519	0.99801	0.99550	0.98652	0.96112
	T = 0.85	0.98328	0.98453	0.96133	0.94294	0.85862
	T = 0.88	0.95141	0.93918	0.85193	0.84994	0.67920
	T = 0.90	0.89540	0.84775	0.70940	0.72392	0.48192
	T = 0.95	0.47816	0.09540	0.05966	0.03730	0.01462
**DFCV-2**	T = 0.70	0.99989	1	1	0.99905	0.99613
	T = 0.75	0.99926	1	1	0.99728	0.98923
	T = 0.80	0.99822	0.99832	0.99613	0.98850	0.96426
	T = 0.85	0.98129	0.98390	0.95517	0.94587	0.85454
	T = 0.88	0.94681	0.93291	0.85203	0.85757	0.65956
	T = 0.90	0.89498	0.84190	0.68401	0.73281	0.45256
	T = 0.95	0.44482	0.07356	0.04179	0.03113	0.01400
**DFCV-3**	T = 0.70	0.99905	0.99905	1	0.99989	0.99853
	T = 0.75	0.99895	0.99895	0.99916	0.99874	0.99362
	T = 0.80	0.99728	0.99770	0.99665	0.99090	0.97126
	T = 0.85	0.98495	0.97857	0.96917	0.94440	0.85464
	T = 0.88	0.96165	0.93166	0.86854	0.83699	0.68317
	T = 0.90	0.91974	0.83479	0.70752	0.70961	0.47513
	T = 0.95	0.47450	0.08317	0.05057	0.03312	0.01400
**DFCV-4**	T = 0.70	0.99989	1	1	0.99926	0.99937
	T = 0.75	0.99885	1	1	0.99728	0.99571
	T = 0.80	0.99791	0.99874	0.99686	0.98787	0.96468
	T = 0.85	0.98390	0.97931	0.94806	0.95308	0.85318
	T = 0.88	0.95224	0.91797	0.83667	0.86050	0.66112
	T = 0.90	0.89592	0.81619	0.67450	0.71765	0.44524
	T = 0.95	0.44357	0.07481	0.04409	0.03448	0.01076
**DFCV-5**	T = 0.70	1	1	1	1	0.99738
	T = 0.75	0.99979	1	1	0.99832	0.99258
	T = 0.80	0.99770	1	0.99759	0.99310	0.97272
	T = 0.85	0.98861	0.98150	0.96394	0.96123	0.87617
	T = 0.88	0.96656	0.93364	0.86603	0.87398	0.69717
	T = 0.90	0.91839	0.84535	0.71525	0.73103	0.47387
	T = 0.95	0.47021	0.08150	0.04660	0.02319	0.01076

## References

[1] Suri J.S., Puvvula A., Biswas M., Majhail M., Saba L., Faa G., Singh I.M., Oberleitner R., Turk M., Chadha P.S. (2020). COVID-19 pathways for brain and heart injury in comorbidity patients: A role of medical imaging and artificial intelligence-based COVID severity classification: A review. Comput. Biol. Med..

[2] Suri J.S., Agarwal S., Gupta S.K., Puvvula A., Biswas M., Saba L. (2021). A narrative review on characterization of acute respiratory distress syndrome in COVID-19-infected lungs using artificial intelligence. Comput. Biol. Med..

[3] Taquet M., Geddes J.R., Husain M., Luciano S., Harrison P.J. (2021). 6-month neurological and psychiatric outcomes in 236 379 survivors of COVID-19: A retrospective cohort study using electronic health records. Lancet Psychiatry.

[4] To K.K.-W., Tsang O.T.-Y., Leung W.-S., Tam A.R., Wu T.-C., Lung D.C., Yip C.C.-Y., Cai J.-P., Chan J.M.-C., Chik T.S.-H. (2020). Temporal profiles of viral load in posterior oropharyngeal saliva samples and serum antibody responses during infection by SARS-CoV-2: An observational cohort study. Lancet Infect. Dis..

[5] Saba L., Gerosa C., Fanni D., Marongiu F., La Nasa G., Caocci G., Barcellona D., Coghe F., Orru G., Coni P. (2020). Molecular pathways triggered by COVID-19 in different organs: ACE2 receptor-expressing cells under attack? A review. Eur. Rev. Med. Pharmacol. Sci..

[6] Viswanathan V., Puvvula A., Jamthikar A.D., Saba L., Johri A.M., Kotsis V., Khanna N.N., Dhanjil S.K., Majhail M., Misra D.P. (2021). Bidirectional link between diabetes mellitus and coronavirus disease 2019 leading to cardiovascular disease: A narrative review. World J. Diabetes.

[7] Cau R., Falaschi Z., Paschè A., Danna P., Arioli R., Arru C.D., Zagaria D., Tricca S., Suri J.S., Kalra M.K. (2021). CT findings of COVID-19 pneumonia in ICU-patients. J. Public Health Res..

[8] Cau R., Pacielli A., Fatemeh H., Vaudano P., Arru C., Crivelli P., Stranieri G., Suri J.S., Mannelli L., Conti M. (2021). Complications in COVID-19 patients: Characteristics of pulmonary embolism. Clin. Imaging.

[9] Congiu T., Demontis R., Cau F., Piras M., Fanni D., Gerosa C., Botta C., Scano A., Chighine A., Faedda E. (2021). Scanning electron microscopy of lung disease due to COVID-19–a case report and a review of the literature. Eur. Rev. Med. Pharmacol. Sci.

[10] Schoene D., Schnekenberg L.G., Pallesen L.P., Barlinn J., Puetz V., Barlinn K., Siepmann T. (2022). Pathophysiology of Cardiac Injury in COVID-19 Patients with Acute Ischaemic Stroke: What Do We Know So Far?—A Review of the Current Literature. Life.

[11] El-Rashidy N., Abdelrazik S., Abuhmed T., Amer E., Ali F., Hu J.-W., El-Sappagh S. (2021). Comprehensive survey of using machine learning in the COVID-19 pandemic. Diagnostics.

[12] Sharma N., Saba L., Khanna N.N., Kalra M.K., Fouda M.M., Suri J.S. (2022). Segmentation-Based Classification Deep Learning Model Embedded with Explainable AI for COVID-19 Detection in Chest X-ray Scans. J. Diagn..

[13] Khanna N.N., Maindarkar M., Puvvula A., Paul S., Bhagawati M., Ahluwalia P., Ruzsa Z., Sharma A., Munjral S., Kolluri R. (2022). Vascular Implications of COVID-19: Role of Radiological Imaging, Artificial Intelligence, and Tissue Characterization: A Special Report. J. Cardiovasc. Dev. Dis..

[14] Chatburn R.L., Branson R.D. (2022). Shortages and Vulnerabilities of Hospital Oxygen Systems. Respir. Care.

[15] Mahase E. (2022). COVID-19: Oxygen shortages two years into pandemic highlight pre-covid failures, says WHO. Br. Med. J. Publ. Group.

[16] DePuccio M.J., Gaughan A.A., Shiu-Yee K., McAlearney A.S. (2022). Doctoring from home: Physicians’ perspectives on the advantages of remote care delivery during the COVID-19 pandemic. PLoS ONE.

[17] Cau R., Faa G., Nardi V., Balestrieri A., Puig J., Suri J.S., SanFilippo R., Saba L. (2022). Long-COVID diagnosis: From diagnostic to advanced AI-driven models. Eur. J. Radiol..

[18] Munjral S., Maindarkar M., Ahluwalia P., Puvvula A., Jamthikar A., Jujaray T., Suri N., Paul S., Pathak R., Saba L. (2022). Cardiovascular Risk Stratification in Diabetic Retinopathy via Atherosclerotic Pathway in COVID-19/Non-COVID-19 Frameworks Using Artificial Intelligence Paradigm: A Narrative Review. Diagnostics.

[19] Chandra T.B., Verma K., Singh B.K., Jain D., Netam S.S. (2021). Coronavirus disease (COVID-19) detection in chest X-ray images using majority voting based classifier ensemble. Expert Syst. Appl..

[20] Liang S., Liu H., Gu Y., Guo X., Li H., Li L., Wu Z., Liu M., Tao L. (2021). Fast automated detection of COVID-19 from medical images using convolutional neural networks. Commun. Biol..

[21] Suri J.S., Maindarkar M.A., Paul S., Ahluwalia P., Bhagawati M., Saba L., Faa G., Saxena S., Singh I.M., Chadha P.S. (2022). Deep Learning Paradigm for Cardiovascular Disease/Stroke Risk Stratification in Parkinson’s Disease Affected by COVID-19: A Narrative Review. J. Diagn..

[22] Alzubaidi L., Zhang J., Humaidi A.J., Al-Dujaili A., Duan Y., Al-Shamma O., Santamaría J., Fadhel M.A., Al-Amidie M., Farhan L. (2021). Review of deep learning: Concepts, CNN architectures, challenges, applications, future directions. J. Big Data.

[23] LeCun Y., Bengio Y., Hinton G. (2015). Deep learning. Nature.

[24] Yamashita R., Nishio M., Do R.K.G., Togashi K. (2018). Convolutional neural networks: An overview and application in radiology. Insights Imaging.

[25] Ullah F., Zhang B., Khan R.U. (2019). Image-based service recommendation system: A JPEG-coefficient RFs approach. IEEE Access.

[26] Chen L., Yang F., Yang H. Image-Based Product Recommendation System with Convolutional Neural Networks. Stanford University. http://cs231n.stanford.edu/reports/2017/pdfs/105.pdf:2017.

[27] Tuinhof H., Pirker C., Haltmeier M. Image-based fashion product recommendation with deep learning. Proceedings of the International Conference on Machine Learning, Optimization, and Data Science.

[28] Saba L., Agarwal M., Patrick A., Puvvula A., Gupta S.K., Carriero A., Laird J.R., Kitas G.D., Johri A.M., Balestrieri A. (2021). Six artificial intelligence paradigms for tissue characterisation and classification of non-COVID-19 pneumonia against COVID-19 pneumonia in computed tomography lungs. Int. J. Comput. Assist. Radiol. Surg..

[29] Zimmerman A., Kalra D. (2020). Usefulness of machine learning in COVID-19 for the detection and prognosis of cardiovascular complications. Rev. Cardiovasc. Med..

[30] Hussain L., Nguyen T., Li H., Abbasi A.A., Lone K.J., Zhao Z., Zaib M., Chen A., Duong T.Q. (2020). Machine-learning classification of texture features of portable chest X-ray accurately classifies COVID-19 lung infection. BioMedical Eng. OnLine.

[31] Agarwal M., Agarwal S., Saba L., Chabert G.L., Gupta S., Carriero A., Pasche A., Danna P., Mehmedovic A., Faa G. (2022). Eight pruning deep learning models for low storage and high-speed COVID-19 computed tomography lung segmentation and heatmap-based lesion localization: A multicenter study using COVLIAS 2.0. Comput. Biol. Med..

[32] Hasan M.M., Murtaz S.B., Islam M.U., Sadeq M.J., Uddin J. (2022). Robust and efficient COVID-19 detection techniques: A machine learning approach. PLoS ONE.

[33] Yang D., Martinez C., Visuña L., Khandhar H., Bhatt C., Carretero J. (2021). Detection and analysis of COVID-19 in medical images using deep learning techniques. Sci. Rep..

[34] Silva P., Luz E., Silva G., Moreira G., Silva R., Lucio D., Menotti D. (2020). COVID-19 detection in CT images with deep learning: A voting-based scheme and cross-datasets analysis. Inform. Med. Unlocked.

[35] Diaz-Escobar J., Ordóñez-Guillén N.E., Villarreal-Reyes S., Galaviz-Mosqueda A., Kober V., Rivera-Rodriguez R., Rizk J.E.L. (2021). Deep-learning based detection of COVID-19 using lung ultrasound imagery. PLoS ONE.

[36] Teodoro A.A., Silva D.H., Saadi M., Okey O.D., Rosa R.L., Otaibi S.A., Rodríguez D.Z. (2021). An analysis of image features extracted by cnns to design classification models for covid-19 and non-COVID-19. J. Signal Process. Syst..

[37] Rahaman M.M., Li C., Yao Y., Kulwa F., Rahman M.A., Wang Q., Qi S., Kong F., Zhu X., Zhao X. (2020). Identification of COVID-19 samples from chest X-Ray images using deep learning: A comparison of transfer learning approaches. J. X-Ray Sci. Technol..

[38] Saiz F., Barandiaran I. (2020). COVID-19 detection in chest X-ray images using a deep learning approach.

[39] Samy B., Al-Hamadi A. (2021). Automatic detection of COVID-19 using pruned GLCM-Based texture features and LDCRF classification. Comput. Biol. Med..

[40] Tello-Mijares S., Woo L. (2021). Computed tomography image processing analysis in COVID-19 patient follow-up assessment. J. Healthc. Eng..

[41] Subramaniam U., Subashini M.M., Almakhles D., Karthick A., Manoharan S. (2021). An expert system for COVID-19 infection tracking in lungs using image processing and deep learning techniques. BioMed Res. Int..

[42] Ahsan M., Based M.A., Haider J., Kowalski M. (2021). COVID-19 detection from chest X-ray images using feature fusion and deep learning. Sensors.

[43] Zhou C., Song J., Zhou S., Zhang Z., Xing J. (2021). COVID-19 detection based on image regrouping and ResNet-SVM using chest X-ray images. IEEE Access.

[44] Chowdhury M.E., Rahman T., Khandakar A., Mazhar R., Kadir M.A., Mahbub Z.B., Islam K.R., Khan M.S., Iqbal A., Al Emadi N. (2020). Can AI help in screening viral and COVID-19 pneumonia?. IEEE Access.

[45] Tahir A.M., Chowdhury M.E., Khandakar A., Rahman T., Qiblawey Y., Khurshid U., Kiranyaz S., Ibtehaz N., Rahman M.S., Al-Maadeed S. (2021). COVID-19 infection localization and severity grading from chest X-ray images. Comput. Biol. Med..

[46] Othman M., Zain N.M., Paidi Z., Pauzi F.A. (2021). Framework of Health Recommender System for COVID-19 Self-assessment and Treatments: A Case Study in Malaysia. Int. J. Comput. Sci. Netw. Secur..

[47] Nilashi M., Asadi S., Abumalloh R.A., Samad S., Ibrahim O. (2020). Intelligent recommender systems in the COVID-19 outbreak: The case of wearable healthcare devices. J. Soft Comput. Decis. Support Syst..

[48] Asabere N.Y., Acakpovi A., Ofori E.K., Torgby W., Kuuboore M., Lawson G., Adjaloko E. (2020). SARPPIC: Exploiting COVID-19 Contact Tracing Recommendation through Social Awareness. Comput. Math. Methods Med..

[49] Deng F., Ren P., Qin Z., Huang G., Qin Z. (2018). Leveraging Image Visual Features in Content-Based Recommender System. Sci. Program..

[50] Sulthana A.R., Gupta M., Subramanian S., Mirza S. (2020). Improvising the performance of image-based recommendation system using convolution neural networks and deep learning. Soft Comput..

[51] Geng X., Zhang H., Bian J., Chua T.-S. Learning image and user features for recommendation in social networks. Proceedings of the IEEE International Conference on Computer Vision.

[52] Kuanr M., Mohapatra P. (2021). Recent challenges in recommender systems: A survey. Progress in Advanced Computing and Intelligent Engineering.

[53] Walek B., Spackova P. Content-based recommender system for online stores using expert system. Proceedings of the 2018 IEEE First International Conference on Artificial Intelligence and Knowledge Engineering (AIKE).

[54] Pal A., Parhi P., Aggarwal M. An improved content based collaborative filtering algorithm for movie recommendations. Proceedings of the 2017 Tenth International Conference on Contemporary Computing (IC3).

[55] Yu C., Tang Q., Liu Z., Dong B., Wei Z. A recommender system for ordering platform based on an improved collaborative filtering algorithm. Proceedings of the 2018 International Conference on Audio, Language and Image Processing (ICALIP).

[56] Shakirova E. Collaborative filtering for music recommender system. Proceedings of the 2017 IEEE Conference of Russian Young Researchers in Electrical and Electronic Engineering (EIConRus).

[57] Devika R., Subramaniyaswamy V. A novel model for hospital recommender system using hybrid filtering and big data techniques. Proceedings of the 2018 2nd International Conference on I-SMAC (IoT in Social, Mobile, Analytics and Cloud) (I-SMAC) I-SMAC (IoT in Social, Mobile, Analytics and Cloud)(I-SMAC).

[58] Kbaier M.E.B.H., Masri H., Krichen S. A personalized hybrid tourism recommender system. Proceedings of the 2017 IEEE/ACS 14th International Conference on Computer Systems and Applications (AICCSA).

[59] Subbotin S., Gladkova O., Parkhomenko A. Knowledge-based recommendation system for embedded systems platform-oriented design. Proceedings of the 2018 IEEE 13th International Scientific and Technical Conference on Computer Sciences and Information Technologies (CSIT).

[60] Wonoseto M.G., Rosmansyah Y. Knowledge based recommender system and web 2.0 to enhance learning model in junior high school. Proceedings of the 2017 International Conference on Information Technology Systems and Innovation (ICITSI).

[61] Wiesner M., Pfeifer D. (2014). Health recommender systems: Concepts, requirements, technical basics and challenges. Int. J. Environ. Res. Public Health.

[62] Kuanr M., Mohapatra P., Piri J. Health recommender system for cervical cancer prognosis in women. Proceedings of the 2021 6th International Conference on Inventive Computation Technologies (ICICT).

[63] Tarasenko A.O., Yakimov Y.V. (2020). Convolutional neural networks for image classification.

[64] He K., Zhang X., Ren S., Sun J. Deep residual learning for image recognition. Proceedings of the IEEE Conference on Computer Vision and Pattern Recognition.

[65] Pathak Y., Shukla P.K., Tiwari A., Stalin S., Singh S. (2020). Deep transfer learning based classification model for COVID-19 disease. J. Irbm.

[66] Suri J.S., Agarwal S., Elavarthi P., Pathak R., Ketireddy V., Columbu M., Saba L., Gupta S.K., Faa G., Singh I.M. (2021). Inter-Variability Study of COVLIAS 1.0: Hybrid Deep Learning Models for COVID-19 Lung Segmentation in Computed Tomography. J. Diagn..

[67] Skandha S.S., Nicolaides A., Gupta S.K., Koppula V.K., Saba L., Johri A.M., Kalra M.S., Suri J.S. (2021). A hybrid deep learning paradigm for carotid plaque tissue characterization and its validation in multicenter cohorts using a supercomputer framework. J. Comput. Biol. Med..

[68] Chu Y., Yue X., Yu L., Sergei M., Wang Z. (2020). Automatic image captioning based on ResNet50 and LSTM with soft attention. Wirel. Commun. Mob. Comput..

[69] Zhang Q. (2022). A novel ResNet101 model based on dense dilated convolution for image classification. SN Appl. Sci..

[70] Pustokhin D.A., Pustokhina I.V., Dinh P.N., Phan S.V., Nguyen G.N., Joshi G.P. (2020). An effective deep residual network based class attention layer with bidirectional LSTM for diagnosis and classification of COVID-19. J. Appl. Stat..

[71] Jiang Z.-P., Liu Y.-Y., Shao Z.-E., Huang K.-W. (2021). An Improved VGG16 Model for Pneumonia Image Classification. Appl. Sci..

[72] Mateen M., Wen J., Song S., Huang Z. (2018). Fundus image classification using VGG-19 architecture with PCA and SVD. Symmetry.

[73] Simonyan K., Zisserman A. (2014). Very deep convolutional networks for large-scale image recognition. arXiv.

[74] Kaur S., Aggarwal D. (2013). Image content based retrieval system using cosine similarity for skin disease images. Adv. Comput. Sci. Int. J..

[75] Kuppili V., Biswas M., Edla D.R., Prasad K.R., Suri J.S. (2018). A mechanics-based similarity measure for text classification in machine learning paradigm. IEEE Trans. Emerg. Top. Comput. Intell..

[76] Khoshdeli M., Cong R., Parvin B. Detection of nuclei in H&E stained sections using convolutional neural networks. Proceedings of the 2017 IEEE EMBS International Conference on Biomedical & Health Informatics (BHI).

[77] Jain P.K., Sharma N., Saba L., Paraskevas K.I., Kalra M.K., Johri A., Laird J.R., Nicolaides A.N., Suri J.S. (2021). Unseen artificial intelligence—Deep learning paradigm for segmentation of low atherosclerotic plaque in carotid ultrasound: A multicenter cardiovascular study. J. Diagn..

[78] Sanagala S.S., Nicolaides A., Gupta S.K., Koppula V.K., Saba L., Agarwal S., Johri A.M., Kalra M.S., Suri J.S. (2021). Ten Fast Transfer Learning Models for Carotid Ultrasound Plaque Tissue Characterization in Augmentation Framework Embedded with Heatmaps for Stroke Risk Stratification. J. Diagn..

[79] Agarwal M., Saba L., Gupta S.K., Johri A.M., Khanna N.N., Mavrogeni S., Laird J.R., Pareek G., Miner M., Sfikakis P.P. (2021). Wilson disease tissue classification and characterization using seven artificial intelligence models embedded with 3D optimization paradigm on a weak training brain magnetic resonance imaging datasets: A supercomputer application. J. Med. Biol. Eng. Comput..

[80] DeTone D., Malisiewicz T., Rabinovich A. Superpoint: Self-supervised interest point detection and description. Proceedings of the IEEE Conference on Computer Vision and Pattern Recognition Workshops.

[81] Rahman T., Khandakar A., Qiblawey Y., Tahir A., Kiranyaz S., Kashem S.B.A., Islam M.T., Al Maadeed S., Zughaier S.M., Khan M.S. (2021). Exploring the effect of image enhancement techniques on COVID-19 detection using chest X-ray images. Comput. Biol. Med..

[82] Chen T., Wu D., Chen H., Yan W., Yang D., Chen G., Ma K., Xu D., Yu H., Wang H. (2020). Clinical characteristics of 113 deceased patients with coronavirus disease 2019: Retrospective study. J. BMJ.

[83] Dufan W., Gong K., Arru C.D., Homayounieh F., Bizzo B., Buch V., Ren H., Kim K., Neumark N., Xu P. (2020). Severity and consolidation quantification of COVID-19 from CT images using deep learning based on hybrid weak labels. IEEE J. Biomed. Health Inform..

[84] Suri J.S., Agarwal S., Chabert G.L., Carriero A., Paschè A., Danna P.S.C., Saba L., Mehmedović A., Faa G., Singh I.M. (2022). COVLIAS 1.0Lesion vs. MedSeg: An Artificial Intelligence Framework for Automated Lesion Segmentation in COVID-19 Lung Computed Tomography Scans. Diagnostics.

